# Ribozyme-enhanced single-stranded Ago2-processed interfering RNA triggers efficient gene silencing with fewer off-target effects

**DOI:** 10.1038/ncomms9430

**Published:** 2015-10-12

**Authors:** Renfu Shang, Fengjuan Zhang, Beiying Xu, Hairui Xi, Xue Zhang, Weihua Wang, Ligang Wu

**Affiliations:** 1 National Center for Protein Science Shanghai, State Key Laboratory of Molecular Biology, Institute of Biochemistry and Cell Biology, Shanghai Institutes for Biological Sciences, Chinese Academy of Sciences, Shanghai 200031, China; 2 Shanghai Science Research Center, Chinese Academy of Sciences, Shanghai 201204, China; 3 Shanghai Key Laboratory of Molecular Andrology, Institute of Biochemistry and Cell Biology, Shanghai Institutes for Biological Sciences, Chinese Academy of Sciences, Shanghai 200031, China; 4 School of Life Sciences, Shanghai University, Shanghai 200444, China

## Abstract

Short-hairpin RNAs (shRNAs) are widely used to produce small-interfering RNAs (siRNAs) for gene silencing. Here we design an alternative siRNA precursor, named single-stranded, Argonaute 2 (Ago2)-processed interfering RNA (saiRNA), containing a 16–18 bp stem and a loop complementary to the target transcript. The introduction of a self-cleaving ribozyme derived from hepatitis delta virus to the 3′ end of the transcribed saiRNA dramatically improves its silencing activity by generating a short 3′ overhang that facilitates the efficient binding of saiRNA to Ago2. The same ribozyme also enhances the activity of Dicer-dependent shRNAs. Unlike a classical shRNA, the strand-specific cleavage of saiRNA by Ago2 during processing eliminates the passenger strand and prevents the association of siRNA with non-nucleolytic Ago proteins. As a result, off-target effects are reduced. In addition, saiRNA exhibits less competition with the biogenesis of endogenous miRNAs. Therefore, ribozyme-enhanced saiRNA provides a reliable tool for RNA interference applications.

## Abstract (web summary)

Short hairpin RNAs are widely used to produce small interfering RNAs (siRNAs) for gene silencing. Here, the authors show that an alternative siRNA precursor in the presence of a self-cleaving ribozyme has enhanced silencing activity and reduced off-target effects, providing a potential RNAi tool.

## Graphical abstract

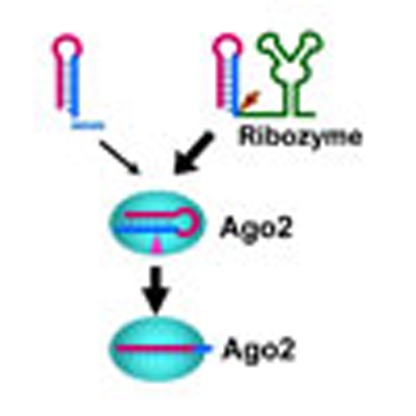

Since its discovery in *Caenorhabditis elegans*, RNA interference (RNAi) has become a powerful tool for silencing target genes in a wide range of cells with great therapeutic potential^1^,^2^,^3^. RNAi is triggered by small-interfering RNA (siRNA), which is a short RNA duplex ∼21-nucleotides (nt) long with a 2 nt overhang at the 3′ end^4^,^5^. Within the cytoplasm, the siRNA duplex binds to Argonaute (Ago) proteins and forms the RNA-induced silencing complex (RISC). The guide strand (the strand perfectly complementary to the target mRNA) and the passenger strand (the strand that has the same sequence as the target mRNA) are subsequently separated within the RISC, and only a single strand is retained. Strand retention is dependent on the thermodynamic stability of base pairing at the 5′ ends of the two strands^6^,^7^. The mature RISC, which contains single-stranded siRNA, recognizes mRNAs that bear perfectly complementary sequences and directs endonucleolytic cleavage at the binding site, triggering the rapid degradation of the entire mRNA^8^,^9^,^10^.

Chemically synthesized siRNAs represent a potent means of inducing RNAi, but the utility of this approach has been limited because of its transient effect in knocking down target genes. MicroRNAs (miRNAs) are endogenous small non-coding RNAs that are very similar to siRNAs. The primary miRNA transcript is usually cleaved by the nuclear microprocessor complex Drosha/DGCR8 to produce a short-hairpin structure (pre-miRNA) that is exported to the cytoplasm by exportin-5. The pre-miRNA is then processed by the cytoplasmic RNase III enzyme Dicer prior to loading into the RISC, which mediates the translational repression and/or mRNA deadenylation of partially complementary mRNAs^11^. In animals, siRNA can be produced from RNA polymerase II (pol II) promoters by substituting the miRNA sequence in a miRNA gene with siRNA^12^,^13^ or from RNA pol III promoters by transcribing a pre-miRNA-like short-hairpin RNA (shRNA)^14^,^15^.

Although RNAi has immense potential in gene silencing for both *in vitro* and *in vivo* applications, reservations have arisen. Of these concerns is the toxicity caused by off-target effects resulting from the partial base pairing of siRNAs with mRNAs and the downregulation of many unintended targets via a miRNA-like mechanism^16^,^17^,^18^,^19^. Furthermore, shRNAs can disrupt endogenous miRNA function by competing for protein factors essential for miRNA biogenesis and functions, such as exportin-5, Dicer and Ago proteins, leading to the de-repression of many miRNA-regulated genes^20^,^21^.

A typical shRNA usually contains a >4 nt loop and a >19 bp stem region. shRNAs with a stem shorter than 19 bp are not efficiently recognized and processed by Dicer^22^. However, a chemically synthesized short-stem shRNA (sshRNA) with a 19 bp stem and 2 nt loop was reported to be particularly potent in silencing^23^; in this case, processing is Dicer independent. The discoveries of multiple non-canonical miRNA biogenesis pathways raise the possibility of using alternative pathways for siRNA production in cells^24^,^25^,^26^,^27^,^28^. For example, the precursor of miR-451 (pre-miR-451) is produced by Drosha/DGCR8 cleavage and binds to Ago2 directly without Dicer processing. Ago2 cleaves in the middle of the 3′ strand of pre-miR-451, and the resulting intermediate is further trimmed by PARN to generate mature miR-451 (refs 26, 27, 28, 29). Pre-miR-451-like shRNAs have been ectopically expressed by an RNA pol III promoter. However, the silencing activity of such constructs was generally weaker compared with classical Dicer-dependent shRNA^30^,^31^,^32^,^33^,^34^,^35^,^36^,^37^.

Herein, we report our investigation of the processing efficiency of shRNAs with different structural features, their impacts on the selective association of siRNA with Ago proteins, and the on-target and off-target effects of RNAi. We find that siRNA precursors can be divided into three categories with different processing patterns and silencing activities; inclusion into these categories is mainly determined by stem length, which influences selective association with and processing by Dicer or Ago2. Interestingly, the 3′ overhang length of short-stem shRNAs also has a substantial impact on its association with Ago2. We have further designed a new siRNA precursor, named single-stranded, Ago2-processed interfering RNA (saiRNA), which is composed of a 16–18 bp stem, a loop complementary to the target transcript and an engineered hepatitis delta virus (HDV) ribozyme at the 3′ end, which generates a short 3′ overhang and enhances silencing activity. The ribozyme-enhanced saiRNA has a higher knockdown efficiency, induces fewer off-target effects and competes less with endogenous miRNAs than shRNAs, and thus represents an excellent alternative tool for RNAi applications.

## Results

### Three categories of shRNA with divergent processing pathways

**Figure 1:**
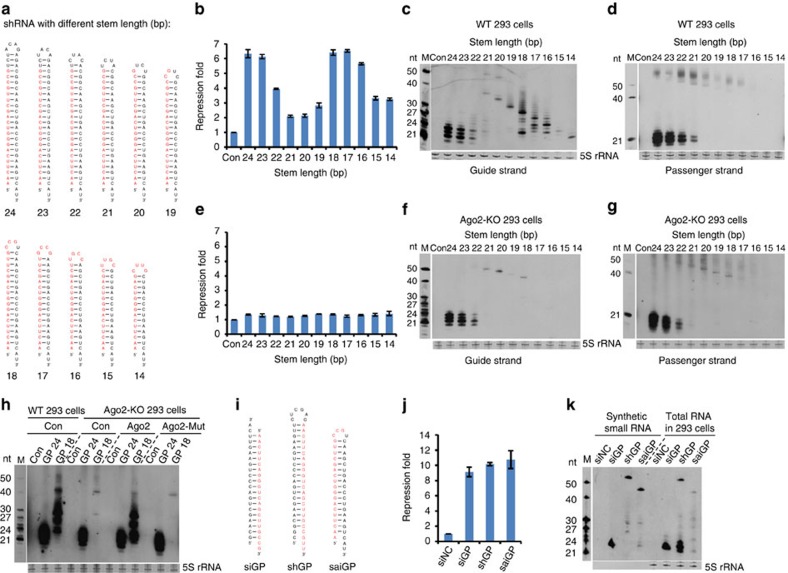
Three categories of shRNAs with different processing patterns and silencing activities. (**a**) Structures of shRNAs with different stem lengths. Nucleotides marked in red represent the guide strand that is complementary to the target sequence. In cases where the stems are shorter than 21 bp, the sequences of the guide strands extend into the loop regions. (**b**) Knockdown efficiency of shRNAs in **a** measured by luciferase assays. The magnitude of repression was calculated based on the expression of a firefly luciferase reporter containing a target sequence perfectly complementary to the guide strand of siGP in its 3′ UTR in the presence versus the absence of each shRNA. A non-target Renilla luciferase reporter served as a transfection control. *R*=1 corresponds to no repression. (**c**,**d**) Processing of shRNAs detected by Northern blotting. Total RNA from HEK293 cells transfected with shRNA in **a** was separated on a 20% denaturing polyacrylamide gel and probed with DNA oligos complementary to the guide (**c**) or passenger (**d**) strand of shRNAs. 5S rRNA served as an internal control. RNA size markers (M) are shown on the left. (**e**) Knockdown efficiency of shRNAs in **a** measured by luciferase assay in Ago2-KO HEK293 cells. (**f**,**g**) Processing of shRNAs in **a** in Ago2-KO HEK293 cells detected by Northern blotting. (**h**) Dependence of Ago2 slicing activity on the processing of short-stem shRNA by compensation assay. shGPs with 24 or 18 bp stems in **a** were cotransfected into wild-type or Ago2-KO HEK293 cells with plasmids expressing a control protein (lacZ), wild-type or cleavage-deficient Ago2. Total RNA was extracted 24 h after transfection and probed for siGP expression by Northern blotting. (**i**) Structure of chemically synthesized siGP, shGP and saiGP. The guide strand is marked in red. (**j**) Knockdown efficiency of the synthesized siRNA precursor in **i** by luciferase assay as in **b**. (**k**) Processing of the synthesized siRNA precursor in **i**. Total RNA from HEK293 cells transfected with equal amounts of synthetic siGP, shGP and saiGP was detected by Northern blotting. All the error bars represent the s.d. of three independent measurements.

To systemically investigate structural influences on the processing of shRNA, we designed a series of shRNAs targeting EGFP with stem lengths ranging from 14 to 24 bp and loops containing 4 or 7 nt (Fig. 1a; Supplementary Fig. 1a). Because base pairing of the first nucleotide at the 5′ end of the siRNA with the target mRNA is not required for the cleavage activity of siRNA, we fixed the first nucleotide of the shRNA sequence as adenine (A) in all constructs. This design not only facilitates efficient transcription initiation by RNA pol III promoter H1 but also enhances the loading of siRNA into the RISC, as a 5′ A or U has the highest affinity for recognition by Ago proteins^38^. The nucleotide at the corresponding site on the 3′ strand of the shRNA was designed as a C or A to avoid base pairing with the 5′ A; such a mismatch has been shown to increase the selective incorporation of the guide strand into the RISC^6^,^7^. Importantly, unlike the constructs of Dicer-dependent classical shRNA used in previous studies, each shRNA was designed so that the stem and loop regions contained as many nucleotides complementary to the 21 nt target sequence as possible, starting from the second nucleotide of the shRNA transcript to the last nucleotide of the loop (Fig. 1a). For example, in an shRNA with a 17 bp stem and a 4 nt loop, the 5′ A is followed by 16 nt in the stem and a consecutive 4 nt in the loop that are complementary to the target sequence (Fig. 1a). Surprisingly, the silencing activity of shRNAs with different stem lengths showed a striking two-peak pattern (Fig. 1b); this was also observed in shRNAs with 7 nt loops (Supplementary Fig. 1b,c). The first peak consisted of shRNAs with stem lengths ranging from 22 to 24 bp, and the second was from 16 to 18 bp. In correlation with their silencing activities, Northern blotting analysis showed that the processing patterns of the shRNAs could be divided into three groups (Fig. 1c). shRNAs with 22–24 bp stems were predominantly processed into siRNAs with lengths of 21–24 nt, typical of Dicer-cleaved products. By contrast, shRNAs with 16–18 bp stems generated more heterogeneous siRNAs (<25 nt) and processing intermediates with lengths longer than 24 nt. Interestingly, shRNAs with 19–20 bp stems only produced some partially processed intermediates longer than 30 nt, but no further trimmed shorter siRNAs (Fig. 1c). The weaker signals observed for the siRNA precursors were partially due to the inhibitory effect of probe hybridization by stable secondary structures such as long hairpins and their partially processed intermediates. This stem-length-dependent two-peak pattern of shRNA silencing was not observed in previous studies^36^,^37^, possibly because the nucleotide sequence located in the loop was not designed to base pair with the target sequence. As a consequence, although the classically designed shRNA with a stem shorter than 19 bp was still processed, the 3′ portion of the derived siRNA could not base pair with its target, resulting in significantly reduced silencing activity.

Interestingly, shRNAs with stems >21 bp produced siRNAs from both strands, whereas shRNAs with stems <19 bp only produced siRNAs corresponding to the 5′ strand (Fig. 1c,d), a pattern similar to pre-miR-451. When the shRNAs were transfected into Ago2-KO HEK293 cells, no repression was observed, indicating that the processing and/or silencing activity of shRNAs is Ago2 dependent (Fig. 1e). Northern blotting analysis showed that shRNAs with stems longer than 21 bp were efficiently processed into siRNAs despite the absence of Ago2. The processed products of shRNAs with stems shorter than 21 bp were almost completely absent, highlighting their dependence on Ago2 (Fig. 1f,g). Furthermore, only the shRNAs with the shorter stem (GP18), but not that with the longer stem (GP24), regained its mature products when a wild-type Ago2, but not an Ago2 slicing mutant, was supplied in Ago2-KO 293 cells (Fig. 1h).

We further analysed the association of different shRNAs with Dicer and Ago2 by co-immunoprecipitation (co-IP) of shRNAs with either a slicing-deficient Dicer (D1320A/D1709A, this mutant is still capable of forming dimers with endogenous wild-type Dicer and thus can pull-down some of the processed siRNAs associated with wild-type Dicer) or an Ago2 (D597A) mutant. Interestingly, we found that shRNA with 19–20 bp stem can be bound by both Dicer and Ago2 (Supplementary Fig. 1d,e) but cannot be processed efficiently by Dicer cleavage (Fig. 1f) or unwinding and trimming after Ago2 slicing in the middle of the 3′ strand (Fig. 1c). These results suggest that the reduced silencing activity observed for shRNAs with 19–20 bp stems is a consequence of less efficient processing by both Dicer-dependent and Ago2-dependent pathways. Loop size did not have a significant impact on the processing patterns of shRNAs with 18 bp stems, even though shRNAs with shorter loops had slightly greater silencing efficiency (Supplementary Fig. 1f–h), possibly because shorter loops are less susceptible to degradation by endonuclease cleavage within cells^23^,^30^. To distinguish the siRNA precursor with a stem of 16–18 bp from classical Dicer-dependent shRNAs, we refer to the former as saiRNA.

### A 3′ ribozyme enhances the activity of saiRNA and shRNA

To further compare saiRNAs with classical shRNAs, we constructed shRNAs following the design guidelines of commonly used vectors such as pLKO.1 (ref. 39). These vectors contain a 6 nt loop and a 21 bp stem, with the guide strand located in the 3′ arm of the stem. These shRNAs produced higher levels of siRNAs and mediated more efficient silencing than their counterparts with guide strands located in the 5′ arm of the stem (Supplementary Fig. 2a–d). Although saiRNAs exhibited silencing activity similar to those of shRNAs containing guide strands located in the 5′ arms of stems, they were less efficient than shRNAs with guide strands located in the 3′ arms of stems. However, when we compared chemically synthesized siRNA precursors by transfecting cells with the same concentrations, the silencing activity of saiRNAs (saiGP or saip53) was comparable to or even more efficient than that of their shRNA counterparts (Fig. 1i,j; Supplementary Fig. 2e,f); the siRNAs processed from saiRNAs were significantly less abundant compared with those processed from shRNAs (Fig. 1k; Supplementary Fig. 2g). These observations were in disagreement with results comparing shRNAs and saiRNAs transcribed by the RNA pol III promoter. A possible explanation for this discrepancy is that RNA pol III-transcribed products usually contain four to six uridines at the 3′ end^40^, much longer than the 2 nt 3′ overhang present in chemically synthesized siRNA or shRNA.

**Figure 2:**
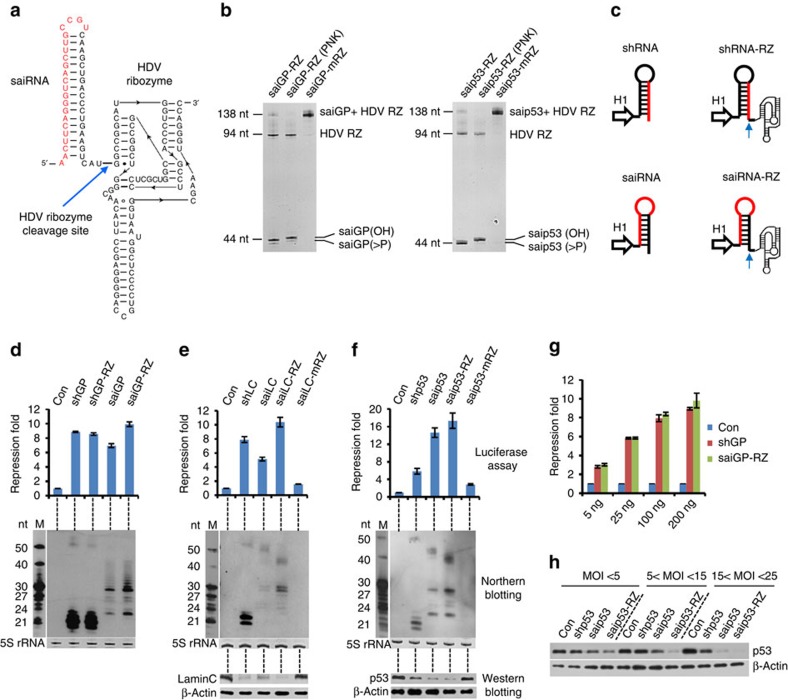
Cleavage at the 3′ end of the siRNA precursor by the HDV ribozyme enhances its expression and knockdown efficiency. (**a**) Secondary structure of saiRNA with HDV ribozyme at the 3′ end. The left part represents the saiRNA, with the guide sequence in red. The right part represents the HDV ribozyme. The blue arrow indicates the HDV ribozyme cleavage site. (**b**) Cleavage of the HDV ribozyme *in vitro*. The saiRNAs fused with a wild-type (saiRNA-RZ) or mutant (saiRNA-mRZ) HDV ribozyme at the 3′ end were transcribed *in vitro* by the T7 RNA polymerase. The transcripts were treated with T4 PNK without ATP and then analysed on a 20% denaturing polyacrylamide gel by ethidium bromide (EB) staining. (**c**) Schematic diagram of the shRNA and saiRNA with or without the HDV ribozyme (HDV-RZ) downstream of the 3′ end of the siRNA precursor. Expression of the siRNA precursors in mammalian cells was driven by an H1 promoter. The blue arrow indicates the cleavage site of HDV-RZ, and nucleotides marked in red represent the guide strand. (**d**) Knockdown efficiency and processing of shGP and saiGP transcribed by the H1 promoter as described in **c**. (**e**,**f**) Knockdown efficiency and processing of shRNA and saiRNA targeting the *laminC (LC)* and *P53* genes in HEK293 cells. Luciferase and Northern blotting assays were performed as in **d**. Changes in protein levels on siRNA expression were determined by western blotting assays with antibodies recognizing laminC or p53. β-actin served as the loading control. (**g**) Effect of transfection dosages on the repression activity of shGP and saiGP-RZ. (**h**) Knockdown efficiency of the endogenous *P53* gene by shRNA or saiRNA stably expressed in HEK293 cells transduced with lentiviral vectors. HEK293 cells were transduced with lentivirus encoding shp53, saip53 or saip53-RZ at different MOIs and selected by puromycin for 6 days. Expression of the *P53* gene was measured by western blotting as in **f**. All the error bars represent the s.d. of three independent measurements.

One method used to produce transcribed RNA with a defined 3′ end is to introduce a ribozyme with a self-cleavage site at the desired position. The HDV ribozyme is an excellent candidate for this purpose because it is able to mediate the self-cleavage of RNA *in vivo* at a precise position upstream of the ribozyme sequence without base pairing with upstream flanking sequences, as has been observed for the hairpin or hammer head ribozyme. We inserted the HDV ribozyme downstream of the 3′ end of the saiGP sequence (Fig. 2a) under the control of a T7 promoter. Following *in vitro* transcription, the inserted HDV ribozyme efficiently cleaved itself out of the transcribed RNA and generated a uniform 3′ overhang (Fig. 2b). A single-nucleotide mutation (C75U) at the catalytic site of the ribozyme completely abolished its cleavage activity and resulted in a single large saiRNA-ribozyme fusion product 138 nt in length. The saiRNA cleaved by the HDV ribozyme contained a 2′, 3′-cyclic phosphate^41^,^42^,^43^. This structure can be converted into a hydroxyl group by treatment with T4 polynucleotide kinase (PNK) and causes slower migration of the corresponding RNA molecule on a denaturing PAGE gel (Fig. 2b).

Next, we fused the HDV ribozyme downstream of the 3′ end of saiRNA in a mammalian expression vector driven by the H1 promoter (saiGP-RZ; Fig. 2c). The silencing efficiency of saiGP improved by >40%, resulting in superior silencing activity compared with that of shGP (Fig. 2d). Northern blotting analysis demonstrated that saiGP containing the HDV ribozyme produced a significantly increased amount of both siGP and the Ago2-cleaved intermediates, suggesting an increase in the stability of saiRNA or its association with Ago2 (Fig. 2d). Adding an HDV ribozyme also improved the repression efficiency of another saiRNA targeting the human *LaminC* gene by 100% (Fig. 2e). Even highly efficient saiRNAs, such as saip53, can be further improved through the addition of an HDV ribozyme (Fig. 2f). As expected, these improvements in silencing activity were consistent with an increased abundance of siRNAs (Fig. 2d–f). When HDV ribozyme cleavage was inactivated, very weak gene silencing was achieved by saiRNAs; both the siRNA products and the Ago2-cleavage intermediates were nearly absent (saiLC-mRZ and saip53-mRZ in Fig. 2e,f), indicating that this improvement was specifically dependent on the catalytic activity of the HDV ribozyme. Intriguingly, the insertion of the same HDV ribozyme also improved the repression efficiency of shRNAs, especially at lower transfection dosage (Supplementary Fig. 2h–k). However, the effects were less pronounced than that observed in saiRNAs, a difference likely due to intrinsic divergence in the processing pathways of saiRNAs (Ago2 dependent) and shRNAs (Dicer dependent). The shorter 3′ overhang may be preferred, but not essential for the recognition and processing of shRNAs by Dicer.

saiRNA with a 3′ HDV ribozyme exhibited robust performance in several common human cell lines derived from different tissues, including HeLa, Hep3B, Huh7, RKO and HCT116 cells (Supplementary Fig. 3a–i). We further designed 20 pairs of shRNA and saiRNA-RZ molecules targeting 4 different genes: *SCAP*, *EGFR*, *Firefly luciferase* and *P53.* Quantitative real time-PCR (RT-qPCR) indicated that ribozyme-enhanced saiRNAs were very potent in silencing endogenous genes, with efficiency comparable to that of classical shRNAs (Supplementary Fig. 4a–d).

The much higher concentration of siRNAs generated from shRNAs compared with that generated from saiRNAs in cells raised the possibility that the saturation of RISC proteins may mask differences in their silencing activity. To exclude such a possibility, cells were transfected with different amounts (5–200 ng) of plasmids encoding saiRNA-RZ or shRNA. As silencing activity increased with the amount of plasmid transfected, the relative repression ratio between shRNA and saiRNA-RZ remained constant, suggesting that the conditions used for the transfection experiment did not lead to the saturation of cellular proteins for RNAi (Fig. 2g; Supplementary Fig. 4e). Furthermore, we investigated silencing activity on endogenous genes by transducing HEK293 cells with lentivirus vectors (Supplementary Fig. 4f). Again, the lentiviral saiRNA-RZ vectors showed similar or even higher efficiencies than their shRNA counterparts in knocking down endogenous genes (Fig. 2h; Supplementary Fig. 4g,h). The lentivirus bearing saip53-RZ was very effective in repressing endogenous p53 protein expression at different multiplicity of infection (MOI), with a nearly 95% knockdown of p53 protein at 15<MOI<25 (Fig. 2h).

### Ago2 binds selectively to saiRNA with a short 3′ overhang

**Figure 3:**
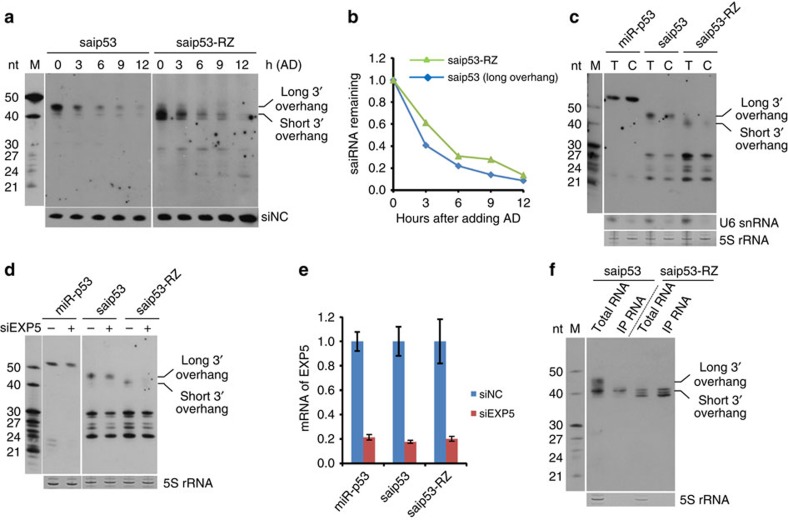
The short 3′ overhang generated by HDV ribozyme cleavage facilitates the efficient binding of saiRNA by Ago2. (**a**) Half-life of saip53 precursors with long or short 3′ overhangs. At 12 h post transfection, 2.5 μg ml^−1^ actinomycin D (AD) was added to Ago2-KO 293 cells to terminate RNA pol III transcription. Cells were harvested at different time points for total RNA extraction and Northern blotting analysis. An unrelated synthetic siRNA (siNC) was added into total RNA to serve as a loading control. (**b**) Graphs of the degradation rate of the saip53 precursor in **a**. The intensity of saip53 at the time immediately after AD addition (0 h) was normalized to 1.0. (**c**) Export of the saiRNA precursors to the cytoplasm. The total RNA (T) and cytoplasmic RNA (C) of HEK293 cells transfected with each siRNA precursor expression plasmid were extracted and analysed by Northern blotting. miR-p53 is a sip53 precursor embedded in a miRNA (miR-26b) backbone and transcribed by RNA pol II. (**d**) Export of saiRNA precursors with long or short 3′ overhangs by Exportin-5. The cytoplasmic RNA of HEK293 cells cotransfected with different siRNA precursors and siEXP5 or a control siRNA were extracted and analysed by Northern blotting. (**e**) Knockdown efficiency of endogenous exportin-5 measured by RT-qPCR. The error bars represent the s.d. of three independent measurements. (**f**) Association of Ago2 with different saiRNA precursors. A FLAG-tagged slicing-deficient Ago2 mutant was cotransfected into Ago2-KO 293 cells with saip53 or saip53-RZ. The FLAG-Ago2mut was immunoprecipitated by anti-FLAG beads. The associated saiRNA precursor was detected by Northern blotting.

Next, we investigated why fusing a ribozyme to the 3′ end of saiRNA promotes siRNA production and function. To compare the stability of saiRNA *in vivo*, we treated Ago2-KO 293 cells with actinomycin D to terminate their transcription. We found the decay rate of saip53 to be comparable to that of saip53-RZ (after the HDV ribozyme is removed by self-cleaving, saip53-RZ is shorter than saip53) (Fig. 3a,b). The RNA transcribed by RNA pol III typically contains poly-U at the 3′ end, which serves as a transcriptional termination signal. Cleavage by the HDV ribozyme at the 3′ end of saiRNA not only generated a shorter (2 nt) 3′ overhang, it also generated a 2′, 3′-cyclic phosphate at the 3′ end (Fig. 2b), a unique feature of ribozyme-cleaved RNA products. Our *in vitro* and *in vivo* assays showed that the presence of 2′, 3′-cyclic phosphate had no significant influence on the stability and repression efficiency of saiRNAs transcribed *in vitro* (Supplementary Fig. 5a–d). Moreover, both saip53 and saip53-RZ expressed in cells can be exported to the cytoplasm with similar efficiency (Fig. 3c). Knocking down the endogenous *EXP5* gene, which is responsible for the transportation of pre-miRNA and shRNA, reduced the accumulation of saip53 and saip53-RZ in the cytoplasm (Fig. 3d,e), consistent with a previous *in vitro* study that exportin-5 bound equally to pre-miRNAs with different 3′ overhang lengths^44^. Longer exposure of Northern blots revealed that saiRNAs also produced a minor short isoform approximately the same size as that of saiRNA-RZ (Fig. 3a; Supplementary Fig. 5e,f), which was likely to be the 3′ trimming product of full-length saiRNA. Interestingly, overexpressing an Ago2 mutant (D597A) in cells specifically increased the accumulation of saip53 with short 3′ overhang (compare the size of saip53 in lane 1 in Fig. 3f with that in lane 1 in Fig. 1a). Furthermore, we compared the association of different saiRNAs with Ago2 by transfecting Ago2-KO 293 cells with the FLAG-tagged slicing-deficient Ago2 mutant and performed IP with the FLAG antibody. While ribozyme-cleaved saip53-RZ can be completely bound by Ago2, only saip53 with short 3′ overhang can associate with Ago2, full-length saip53 did not associate with Ago2 (Fig. 3f), suggesting Ago2 binding is dependent on the length of 3′ overhang. Taken together, these results indicate that although saiRNA and saiRNA-RZ are present in cells with similar abundance, the inability of saiRNA with long-3′ overhang to bind to Ago2 results in a reduced accumulation of siRNA, thus low silencing activity. Removing the extra sequence via the HDV ribozyme generates a short 3′ overhang that facilitates the efficient association of saiRNA with Ago2 and greatly improves silencing activity.

### saiRNA induces fewer off-target effects than shRNA

Despite the fact that the abundance of siRNAs produced from HDV ribozyme-enhanced saiGP and saiLC was much lower than that from the corresponding shRNAs (Fig. 2d,e), the silencing activities of the saiRNAs were similar or even higher than those of the shRNAs. The amount of siRNA generated from chemically synthesized saiRNA in the cells was three to five fold less than that from shRNA, although siRNA repressed the target gene equally or more efficiently (Fig. 1j,k; Supplementary Fig. 2f,g). These data suggest that the silencing activity per molecule of siRNA derived from saiRNA is significantly greater than that of shRNA, an extraordinary feature for RNAi. Moreover, all four mammalian Ago paralogues can associate with siRNAs. However, two of the non-nucleolytic Ago proteins are much more effective than Ago2 in repressing the expression of partially complementary targets and result in stronger off-target effects. In contrast, Ago2 contribute to more on-target than off-target RNAi effects due to its intrinsic nucleolytic activity^45^. Hence, the selective loading of siRNA into nucleolytic or non-nucleolytic Ago proteins is expected to have a substantial impact on both the efficiency (on-target) and specificity (on-target versus off-target effects) of RNAi. Because the processing of saiRNA is Ago2-cleavage dependent, theoretically, only Ago2-RISC will associate with the siRNA derived from saiRNA. Therefore, the preference for loading small RNAs into Ago2-RISC may result in the greater efficiency of on-target RNAi with reduced off-target effects.

**Figure 4:**
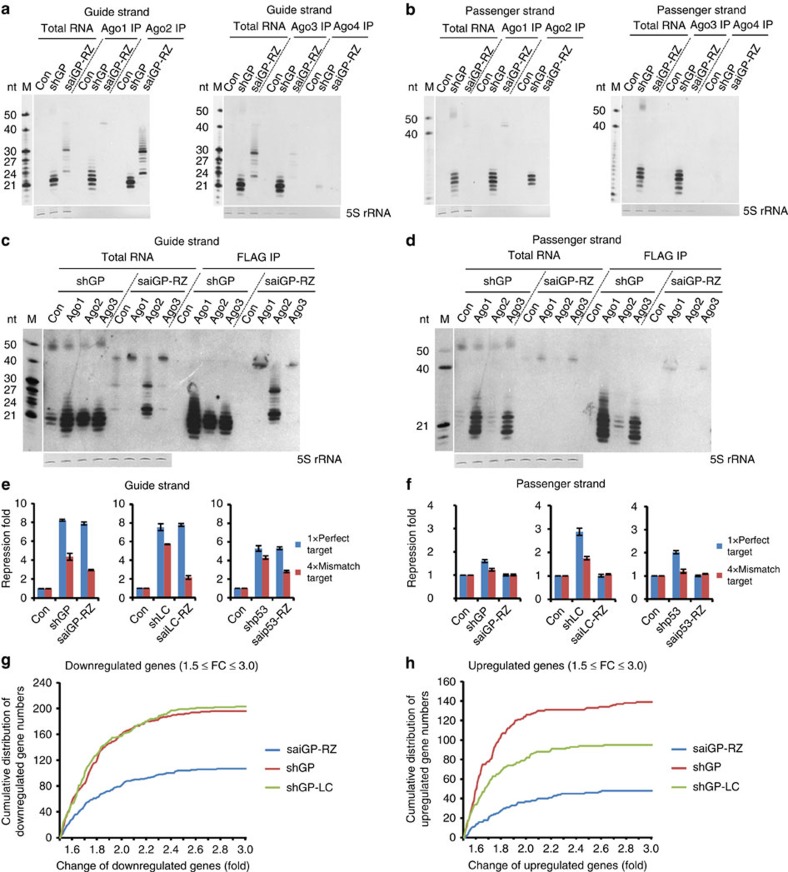
saiRNA induces fewer off-target effects than shRNA. (**a**,**b**) Ago-bound guide strands (**a**) and passenger strands (**b**) generated from shGP and saiGP-RZ. Plasmids encoding shGP or saiGP-RZ or a control plasmid (con) without an shRNA sequence were transfected into HEK293 cells. Endogenous Ago1, 2, 3 and 4 were immunoprecipitated using Ago-specific antibodies. The associated small RNAs were analysed by Northern blotting as in Fig. 1c. (**c**,**d**) Association of guide strands (**c**) or passenger strands (**d**) generated from shGP and saiGP-RZ with ectopically expressed different Ago proteins. Plasmids encoding shGP or saiGP-RZ were cotransfected with plasmids expressing FLAG-tagged Ago1, 2, 3 or control protein (firefly luciferase) into HEK293 cells. Anti-FLAG IP followed by Northern blotting was performed to determine the associated small RNAs. (**e**,**f**) Off-target effects caused by guide strands (**e**) and passenger strands (**f**) of shRNA and saiRNA-RZ targeting the *EGFP*, *laminC* and *P53* genes. On-target effects of the guide strand from shRNA and saiRNA-RZ were measured using a luciferase gene containing a perfectly complementary target. The results were adjusted to a similar level by reducing the amount of saiRNA-RZ plasmids used in the transfection. Off-target effects due to the guide strands were measured using a luciferase gene containing four target sites partially complementary to the guide strands. Off-target effects of the passenger strands were measured using a luciferase gene bearing a perfectly complementary target or four target sites partially complementary to the passenger strands. The error bars represent the s.d. of three independent measurements. (**g**,**h**) Cumulative distribution of downregulated (**g**) or upregulated (**h**) genes in HEK293 cells transfected with shGP, shGP-LC or saiGP-RZ compared with a control sample without shRNA. The genes expressed with FPKM ⩾10 and a fold change between 1.5 and 3 are plotted.

To validate this hypothesis, we characterized the association of different Ago proteins with siRNAs derived from shRNA or saiRNA via the IP of endogenous Ago proteins by Ago family member-specific antibodies in which a lack of cross-reactivity has been verified (Supplementary Fig. 6a–d). Both the endogenous nucleolytic and non-nucleolytic Agos bound equally efficiently to the guide strand or passenger strand of siRNAs derived from shGP, with the exception of Ago4 which was expressed in the examined cells at a level below detection limits (Fig. 4a,b). Ago2 demonstrated a strong association with siRNA and partially processed saiRNA intermediates (saiGP-RZ). Ago1 bound only unprocessed saiRNA (Fig. 4a), which was consistent with its inability to cleave as endonucleases. Although Ago3 appeared to bind some processed and unprocessed saiRNAs, the amount was at least six times less than that of Ago2 and may have been due to the residual cleavage activity of Ago3 (ref. 46). This binding selectivity was even more obvious when the N-terminal FLAG-tagged Ago1/2/3 proteins ectopically expressed in HEK293 cells were immunoprecipitated (Fig. 4c,d). Overexpression of Ago2 enhanced saiRNA processing, whereas Ago1 and Ago3 overexpression resulted in the accumulation of unprocessed saiRNA precursors and a reduction in siRNAs (Fig. 4c). Given this remarkable difference in Ago-bound products from shRNA or saiRNA, we further compared the on-target and off-target effects of RNAi using luciferase reporters (Supplementary Fig. 6e). Although the on-target repression efficiency was similar, the off-target effects caused by saiRNA-RZ were significantly reduced compared with those caused by shRNA (Fig. 4e).

Off-target effects were induced by the guide strand of siRNA, as well as the passenger strand targeting both perfectly and partially complementary sequences of genes. Designing shRNA using thermodynamic stability rules of 5′ end base pairing can reduce the loading of the passenger strand into the RISC but cannot eliminate this effect entirely (Fig. 4b,d). Notably, no signal of passenger strands derived from saiRNA was detected in the input total RNA or RNAs immunoprecipitated by Ago1/2/3 in Northern blotting assays (Fig. 4b,d). Consistent with these observations, the off-target effects caused by passenger strands of shRNAs targeting *EGFP*, *LaminC* and *P53* were readily detected at various levels by the reporter assay. No off-target effect was detected for any of the corresponding saiRNAs (Fig. 4f). In mammals, many protein-coding genes possess antisense transcripts with important regulatory functions^47^,^48^,^49^; such genes include *p15* (*INK4b*), *BACE1* and *BDNF*^50^,^51^,^52^. We constructed reporter plasmids containing an overlapping region of mRNAs and their antisense transcripts inserted downstream of a firefly luciferase gene in the forward (sense target) or reverse (antisense target) orientation. The shRNAs targeting sense mRNA had a strong influence on the expression of antisense-transcript reporters, whereas their saiRNA counterparts showed no such effect (Supplementary Fig. 6f,g).

For a more comprehensive analysis of the off-target effects on endogenous genes, we performed transcriptome analyses of HEK293 cells transiently transfected with an equal amount of a control vector not expressing small RNA (con), shGP, saiGP-RZ or shGP-LC (an improved shRNA design for generating a more accurate 5′ end of the siRNA)^53^. Northern blotting showed that both shGP and shGP-LC produced much more siGP than saiGP-RZ under the same transfection dosage (Supplementary Fig. 7a), despite similar repression activity on the target gene. In saiRNA-transfected HEK293 cells, significantly fewer genes were downregulated by >1.5-fold compared with shGP- or shGP-LC-transfected cells (Fig. 4g; Table 1; Supplementary Data 1). More than half of the downregulated mRNAs contained at least one target site in their 3′ untranslated region (UTR) that was complementary to the 2–7 nt seed sequence of the major isoforms of the guide strand as determined via deep sequencing of the small RNAs in those cells (Table 1; Supplementary Fig. 7b; Supplementary Data 2). Moreover, a significant portion of the downregulated mRNAs appeared to be targeted by the passenger strand of shGP and shGP-LC (Table 1). Other genes may have been downregulated due to the off-target effects by non-canonical base pairing between siRNAs and target mRNAs or due to secondary effects. To confirm the results of the transcriptome analysis, we randomly selected 14 genes significantly downregulated by off-target effects (Supplementary Fig. 7d) and detected their relative expression level by RT-qPCR in three biological repeats. Good correlation with the transcriptome data was observed (Supplementary Fig. 7e). Cells transfected with a control vector containing only the HDV ribozyme displayed no such changes, indicating that the ribozyme itself had no influence (Supplementary Fig. 7e).

**Figure 5:**
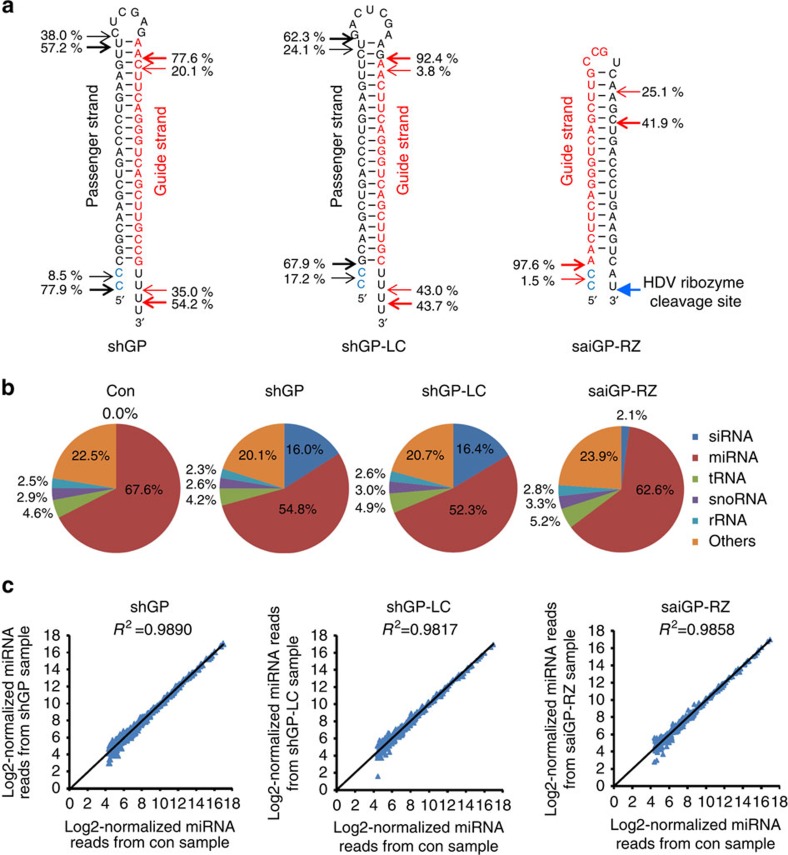
saiRNA exhibits less competition with endogenous miRNAs than does shRNA. (**a**) Processing of shGP, shGP-LC and saiGP-RZ analysed by deep sequencing. Small RNAs from HEK293 cells transfected with plasmids encoding shGP, shGP-LC or saiGP-RZ were subjected to deep-sequencing analysis. The 5′ and 3′ ends of the two most abundant isoforms of the guide strand or passenger strand are labelled with red or black arrows, respectively; relative abundance is indicated next to the arrows. The nucleotides in blue indicate the sequence of the H1 promoter. The blue arrow indicates the HDV ribozyme cleavage site. (**b**) Relative abundance of endogenous small non-coding RNAs and siRNAs in HEK293 cells transfected with shGP, shGP-LC, saiGP-RZ or an empty vector (con) measured by deep sequencing. (**c**) Correlations of the abundance of the top 300 miRNAs in cells transfected with shGP, shGP-LC, saiGP-RZ or an empty vector. Expression levels of individual miRNAs were normalized to the total miRNA counts in each sample. The top 300 miRNAs were used for correlation analysis between the control and shGP, shGP-LC or saiGP-RZ.

The 5′ nucleotide identity in the siRNA is not only related to the efficiency of Ago protein association but also to the off-target effects that depend on the 2–7 nt seed region of the siRNA. Deep sequencing analyses of small RNAs in the cells showed that the guide strands produced from saiGP-RZ and shGP-LC had a homogenous 5′ end, whereas both the guide strands and passenger strands from shGP were highly heterogeneous at their 5′ ends (Fig. 5a; Supplementary Fig. 8a; Supplementary Data 3), which can cause additional off-target effects^53^. Taken together, these results indicate that saiRNA induces fewer off-target effects on endogenous genes than shRNAs or their modified versions.

### saiRNA competes less with endogenous miRNA than does shRNA

Our transcriptome analysis also revealed significantly fewer genes were upregulated >1.5-fold in cells expressing saiGP-RZ compared with cells expressing shGP and shGP-LC (Fig. 4h; Table 1). TargetScan^54^ predictions demonstrated that nearly 80% of these genes contained at least 1 conserved miRNA target site for the 50 most abundant miRNAs (Supplementary Fig. 7c; Supplementary Data 4). Previous studies have shown that the sustained overexpression of shRNA interfered with the processing and exporting of endogenous miRNAs^20^. To qualitatively investigate this possibility, we performed deep sequencing to profile the expression of endogenous small RNAs in HEK293 cells transfected with saiGP-RZ, shGP or shGP-LC. Ectopic expression of shGP or shGP-LC caused a significant decrease in the proportion of miRNAs to total small RNAs, whereas saiGP-RZ had much less influence on miRNA expression (Fig. 5b; Supplementary Data 5). The siRNAs derived from shGP represented >15% of the total small RNAs and were almost seven times more abundant than those derived from saiGP-RZ (Fig. 5b), a finding consistent with our Northern blotting analysis (Fig. 2d). Interestingly, the relative abundance of individual miRNAs compared with total miRNAs did not change in the correlation analysis, suggesting that the decrease in the proportion of total miRNAs on shRNA overexpression is most likely mediated by competition for the general processing and export machinery of miRNAs (Fig. 5c; Supplementary Data 6). A similar observation was made for shRNA and saiRNA targeting *LaminC* (Supplementary Fig. 8b,c). These results indicate that saiRNA has less influence on the biogenesis of endogenous miRNAs, which is consistent with their unique Dicer-independent biogenesis pathway and the lower accumulation of siRNAs associated with Ago proteins.

## Discussion

In this study, we have investigated shRNAs with different structural features and designed a new potent siRNA precursor enhanced by a ribozyme. The siRNA precursors can be divided into three categories according to their processing pathway, which is mainly determined by stem length. Classical shRNAs have a stem longer than 21 bp and can be efficiently recognized and processed by Dicer. shRNAs with a stem of 16–18 bp (saiRNA) are processed in a manner similar to pre-miR-451, which is Ago2 dependent. Interestingly, we found that shRNAs with a stem of medium size (19–20 bp) can associate with both Dicer and Ago2. However, these shRNAs appear to be incompetent substrates for downstream processing by either pathways; 19–20 bp stems are too short for Dicer cleavage and generate intermediates that are too stable for unwinding and trimming to produce functional siRNAs after cleaving by Ago2 at the centre of the 3′ strand (Fig. 1c,f).

In contrast to chemically synthesized saiRNAs with 2 nt 3′ overhangs, which are very potent (Fig. 1j,k), the silencing activity of saiRNAs transcribed by RNA pol III is usually less efficient than that of classical shRNAs^36^,^37^, which hampers the general application of this technology *in vitro* and *in vivo*. Our experiments demonstrate that such discrepancies are caused by the incompetent binding of Ago2 to saiRNAs with long-3′ overhangs (Fig. 3f). In contrast to natural Ago2 substrates with 2 nt 3′ overhangs, nascent saiRNAs produced by RNA pol III typically contain poly-U tails at the 3′ end. Recent studies have shown that the poly-T signal is not always sufficient to mediate transcriptional termination by RNA pol III and may generate even longer 3′ sequences from DNA templates^55^. By introducing a potent HDV ribozyme downstream of the saiRNA, we efficiently remove extra sequences from the 3′ end and produced a short 3′ overhang, which greatly enhance the association of saiRNA with Ago2 (refs 56, 57). This design significantly increases the accumulation of siRNAs and improves the silencing efficiency of saiRNA to a level comparable to or better than that of the classical shRNAs. The molecular details of how Ago2 is able to distinguish the saiRNAs with different 3′ overhang lengths are unclear and worth further investigation. However, we cannot rule out the possibility that such selectivity is assisted by other cofactors that interact with Ago2. Interestingly, the fusing of an HDV ribozyme also improves the silencing activity of Dicer-dependent shRNAs, consistent with the preference of Dicer binding to hairpin RNAs with 2 nt 3′ overhangs^58^. These findings provide a simple and effective method for enhancing the efficiency of both saiRNAs and shRNAs.

**Figure 6:**
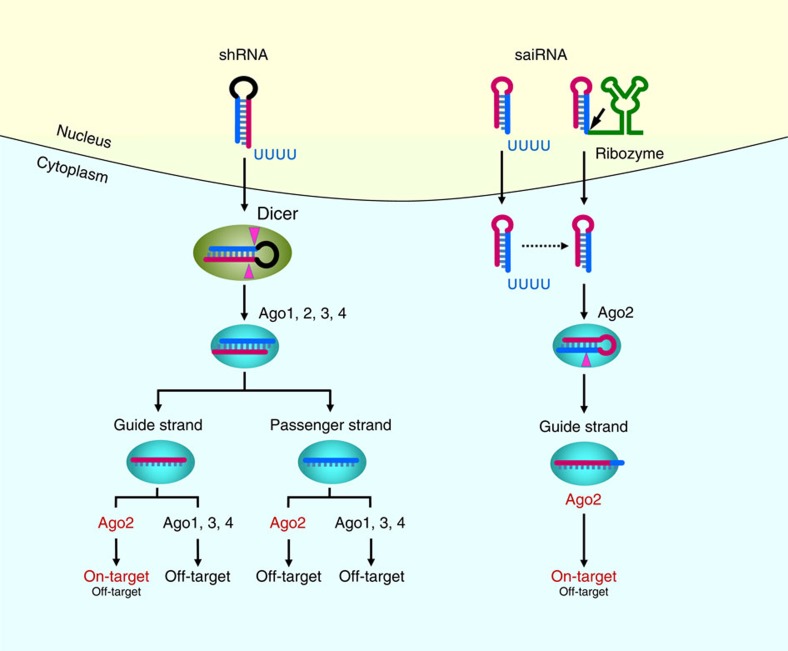
Model of shRNA and saiRNA biogenesis and functions in RNAi. shRNA (stem >20 bp) and saiRNA (stem <19 bp) possess distinct processing mechanisms and patterns of association with Ago proteins. For shRNA, both the guide strand (carmine) and the passenger strand (blue) of the siRNA are produced by Dicer cleavage and are incorporated into all Ago proteins. Both the strands associated with Ago1, 3 and 4 can cause off-target effects. The passenger strand associated with Ago2 not only mediates off-target effects but also cleaves the antisense transcript of target mRNA with possibly unintended consequences. Only the guide strand associated with Ago2 can mediate on-target RNAi (the cleavage of perfectly complementary mRNAs). For saiRNA, the stem region (<19 bp) is too short to be efficiently bound and processed by Dicer. Only the guide strand of saiRNA can be generated by Ago2 cleavage and 3′ trimming, which completely eliminates the off-target effects mediated by the passenger strand. Ago1, 3 and 4 are incapable of processing saiRNA due to their lack of endonucleolytic activity and do not cause any off-target effects. The length of the 3′ overhang is critical for saiRNA loading into Ago2, and only the saiRNAs with short 3′ overhangs can bind to Ago2 efficiently. However, the nascent saiRNA transcribed by RNA pol III typically contains extra nucleotides (such as poly-U tail that serves as a transcriptional termination signal) at the 3′ overhang; these additional nucleotides strongly inhibit the association of saiRNA with Ago2. The fusion of an engineered HDV ribozyme (the hairpin region in green) to the 3′ end of saiRNA efficiently removes the extra nucleotides on the transcript and generates a short 3′ overhang, dramatically improving saiRNA and Ago2 binding and silencing activity.

The specific processing pathway of saiRNA has several advantages that enhance both the efficiency and specificity of RNAi. First, the exclusive association of processed siRNAs derived from saiRNA with Ago2, but not Ago1/3/4, precludes the off-target effects caused by slicer-incompetent Ago proteins. Second, although the guide strand of siRNA with thermodynamically less stable base pairing at the 5′ end is preferentially loaded into the RISC, a significant portion of the passenger strand can still be incorporated into the RISC and mediate off-target effects. By contrast, the strand-specific cleavage of saiRNA by Ago2 during its processing completely eliminates the passenger strand and reduces off-target effects. Importantly, transcriptome analyses have revealed the existence of numerous antisense transcripts that may have important regulatory functions^47^,^48^,^49^. The single-strand feature of saiRNA could be especially useful for studying gene function without the risk of unintentional cleavage of antisense transcripts and lncRNAs mediated by the siRNA passenger strand, which may have unpredictable consequences. Deep-sequencing results show that siRNAs derived from saiRNAs possessed a homogenous 5′ end, further reducing off-target effects caused by the imprecise 5′ end processing of shRNAs. Moreover, small RNA profiling by deep sequencing shows that saiRNA exhibits less competition with the biogenesis of endogenous miRNAs and thus less interference with the expression of miRNA targets. Interestingly, saiRNAs either synthesized chemically or transcribed *in vivo* by RNA pol III generate a lower level of processed single-stranded siRNAs that nonetheless repress target mRNAs as efficiently as shRNAs. This result is likely due to the exclusive association of siRNAs generated from saiRNAs with Ago2 but not the other three non-nucleolytic Ago proteins^32^,^34^. Owing to its higher knockdown efficiency per siRNA molecule, as well as fewer off-target effects and less competition with endogenous miRNA processing, HDV ribozyme-enhanced saiRNA appears to be a powerful RNAi tool for studying gene function (Fig. 6). Although the rules for the design of effective saiRNAs are still elusive, several high-throughput *in vitro* screening methods for highly potent RNAi triggers might be modified to identify the most effective saiRNAs and describe general principles for the design of saiRNAs^59^,^60^.

## Methods

### Plasmids

Plasmids for the expression of shRNA and saiRNA were constructed by inserting annealed DNA oligonucleotides containing shRNA or saiRNA sequences between BamHI/HindIII sites downstream of the H1 promoter derived from the pSilencer 3.1-H1 puro vector (Ambion). The design guidelines of commonly used classical shRNA vectors, such as pLKO.1, can be found from the web site of The RNAi Consortium (TRC) at Broad Institute (http://www.broadinstitute.org/rnai/public/seq/search). For shRNA-RZ or saiRNA-RZ, the HDV ribozyme was amplified by PCR and fused to the 3′ end of the shRNA or saiRNA. The catalytic inactive mutant (C75T) of the HDV ribozyme in saiRNA-mRZ was generated by PCR. To construct lentiviral vectors expressing siRNA precursors, the annealed DNA oligonucleotides encoding shRNA or saiRNA were inserted between the BamHI/XbaI sites and saiRNA-RZ between the BamHI/SphI sites downstream of the H1 or U6 promoter. The guide strand sequence of the siRNA used for the design of the saiRNA in Supplementary Fig. 4a–d was selected using the Dicer-independent miRNA design rules^61^. The luciferase plasmids used in the reporter assays were constructed by inserting annealed DNA oligonucleotides containing siRNA target sequences between NheI/XbaI sites in the 3′ UTR of firefly luciferase^45^,^62^. The reporter plasmids used in Supplementary Fig. 6f,g were constructed by inserting RT-PCR-amplified fragments from target mRNAs (225–2,079 in *p15* mRNA, 659–768 in *BACE1* mRNA and 1,185–1,415 in *BDNF* mRNA) into NheI/XbaI sites in the 3′ UTR of firefly luciferase in forward or reverse orientations. All the primers are listed in Supplementary Methods. The plasmids used for expressing FLAG- or haemagglutanin (HA)-tagged Ago proteins were constructed by inserting RT-PCR-amplified FLAG- or HA-tagged Ago ORF downstream of the CMV promoter^45^.

### RNA oligonucleotides

The RNA oligonucleotides for siGP, shGP, saiGP, sip53, shp53 and saip53 used for transfection were chemically synthesized by Integrated DNA Technologies (IDT). For shRNA and saiRNA, the resuspended oligonucleotides were heated to 95 °C for 5 min and snap-cooled in an ice-water bath to eliminate dimers. For siRNA, 100 μM of the 21 nt single-stranded RNAs in the same volume of annealing buffer was heated to 60–65 °C for 5 min, centrifuged and slowly cooled to room temperature for 30 min to create double-stranded siRNAs. RNA ladders composed of 21, 24, 27, 30, 40 and 50 nt chemically synthesized RNA oligonucleotides that contained the common sequence (5′ AACUUCAGGGUCAGCUUGCCG -3′) were used for probing in the Northern blotting assay. The RNAs used for the self-cleavage assay of the HDV ribozyme were transcribed *in vitro* using the T7 RNA pol from DNA templates containing saiRNA-RZ or saiRNA-mRZ sequences. To remove 2′, 3′-cyclic phosphate from the 3′ end of the saiRNA, *in vitro* transcribed saiRNA-RZ products were treated with T4 PNK (NEB) under conditions deprived of ATP at 37 °C for 2 h. Then, T4 PNK was inactivated by incubating at 65 °C for 20 min. The RNA oligonucleotides are listed in Supplementary Methods.

### Cell culture and reporter assays

HEK293, HeLa, Hep3B, Huh7, RKO and HCT116 cells were obtained from ATCC (Manassas, Virgina, USA) and grown in Dulbecco's modified Eagle's medium (GIBCO) supplemented with 10% FBS. Mycoplasma contaminations were regularly tested for all the cell lines. Transient transfection of the cells with shRNA or saiRNA plasmids (100 ng per well) or RNA oligonucleotides (5 pmol per well) and luciferase plasmids (25 ng per well) was performed in 12-well cell culture plates using Lipofectamine2000 (Invitrogen) according to the manufacturer's protocol. Cells were harvested in 150 μl lysis buffer (Promega) 24 or 48 h post transfection, and firefly and Renilla luciferase activity was measured with a BERTHOLD LB940 instrument using the Dual-Glo luciferase assay system (Promega)^45^.

### Co-immunoprecipitation

HEK293 cells were transiently transfected with pH1-shGP or pH1-saiGP-RZ. After 48 h, the cells were washed with phosphate-buffered saline and harvested in lysis buffer (50 mM Tris-HCl (pH 7.4), 150 mM NaCl, 1 mM EDTA, 0.5 mM dithiothreitol, 0.5% NP-40, 0.1 U μl^−1^ RNase Inhibitor (Fermentas) and 1/100 protease inhibitor cocktail (Sigma)). After rotation at 4 °C for 20 min, each lysate was clarified by centrifugation at 14,000*g* at 4 °C for 15 min. A total of 400 μl supernatant (40 μl saved as input) was mixed with 4 μg antibody specifically recognizing Ago1 (RN028PW, *MBL*), Ago2 (04–642, *Millipore*), Ago3 (39787, *Active Motif*) or Ago4 (05–967, *Millipore*) coupled with Protein A or G beads (Invitrogen) and incubated overnight with gentle rotation at 4 °C. The beads were washed four times with Tris-buffered saline before TRIzol reagent (Takara) was added for RNA extraction. For IP of overexpressed Agos, HEK293 or Ago2-KO 293 cells were cotransfected with plasmids that encoded N-terminally FLAG- or HA-tagged Ago proteins and pH1-shRNA or saiRNA and then immunoprecipitated using the EZview Red Anti-FLAG or HA Affinity Gel (Sigma) as described above.

### Measurement of RNA half-life

To measure the half-life of saiRNA and saiRNA-RZ *in vivo*, Ago2-KO 293 cells were transfected with pH1-saip53 or saip53-RZ plasmids. At 12 h post transfection, 2.5 μg ml^−1^ actinomycin D (Sigma) was added to terminate the transcription of RNA pol III. The cells were harvested at different time points for RNA extraction and Northern blotting analysis. To measure the half-life of saiRNA with terminal 2′, 3′-cyclic phosphate or hydroxyl groups, T7-transcribed saiRNA-RZs treated or untreated with T4 PNK were incubated with Ago2-KO 293 cell lysates in RIPA buffer (50 mM Tris-HCl (pH 7.4), 150 mM NaCl, 1 mM EDTA, 0.5 mM dithiothreitol, 1% NP-40, 0.5% sodium deoxycholate (Sigma), 0.1 U μl^−1^ RNase Inhibitor and 1/100 protease inhibitor cocktail) for different time periods. The total RNA was extracted at different time points using TRIzol-LS reagent (Invitrogen) and was analysed by Northern blotting.

### Northern blotting

To analyse the processing products of shRNA or saiRNA, total cellular RNA transfected with shRNA or saiRNA was extracted using TRIzol reagent. For cytoplasmic RNA preparation, 1 × 10^7^ digested HEK293 cells were centrifuged and resuspended by gently pipetting up and down in 400 μl of ice-cold hypotonic lysis buffer (10 mM Tris-HCl (pH 7.5), 10 mM NaCl, 3 mM MgCl_2_, 0.3% NP-40 and 10% glycerol). The mixture was incubated on ice for 10 min and clarified by centrifugation at 1,000*g* at 4 °C for 3 min. About 250 μl supernatant was mixed with 750 μl Trizol-LS reagent for cytoplasmic RNA extraction^63^. Equal amounts of total RNA (5 μg) were denatured, fractionated by electrophoresis on a 20% polyacrylamide-8 M urea gel at 500 V until bromophenol blue reached the bottom of the gel, and electroblotted and cross-linked onto a nylon membrane (Roche). The blots were probed at 50 °C in DIG Easy Hyb buffer (Roche) with terminally digoxigenin-labelled DNA oligonucleotides overnight and then washed with 2 × SSC and 0.1 × SSC-0.1% SDS buffer at 37 °C. The blots were incubated with an anti-digoxigenin antibody (11093274910, Roche) diluted to 1:20,000 and then CDP-Star (ABI), and the signal was detected on X-ray film. All the uncropped scans of the Northern blots are shown in Supplementary Fig. 9.

### Western blotting

HEK293 cells were harvested and lysed for separation on 10% polyacrylamide-SDS gels and then transferred to a nitrocellulose membrane^62^. The blots were probed for 2 h at room temperature with mouse monoclonal anti-P53 antibodies (P6874, Sigma) diluted to 1:1,000, rabbit polyclonal anti-laminA/C antibodies (BS1446, Bioworld Technology) diluted to 1:500, or mouse monoclonal anti-β-actin antibodies (CW0097, CoWin Biotech) diluted to 1:2,000 and then incubated with a secondary antibody conjugated to horseradish peroxidase (Jackson ImmunoResearch Laboratorie*s*) diluted to 1:5,000. The signal was detected with the Immun-Star HRP chemiluminescence kit (Thermo). All the uncropped scans of the western blots are shown in Supplementary Fig. 9.

### Real-time quantitative PCR

To quantify the knockdown efficiency of shRNA or saiRNA, 1 μg total RNA isolated from HEK293 cells was treated with 1 U DNaseI (Fermentas) and reverse transcribed using M-MLV (Takara) according to the manufacturer's instructions. RT- PCR was performed on a StepOne Plus real-time PCR system (ABI) with Power SYBR Green PCR Master Mix (ABI). The PCR mixtures were heated to 94 °C for 2 min and then subjected to 40 amplification cycles (15 s at 94 °C; 30 s at 60 °C). The data were analysed with StepOne software v2.1 (ABI). The primers used for qPCR are listed in Supplementary Methods.

### Lentiviral vector production and transduction

HIV-1-derived lentiviral particles were produced by transfecting HEK293T cells with pCMVΔR8.91, pCMV-VSV-G and a lentiviral plasmid expressing shRNA or saiRNA using the Sofast reagent (Sunma Biotech Corp) in six-well plates. Cell culture supernatants were collected at 48 and 72 h after transfection and mixed together, followed by filtration through a 0.45-μm filtration membrane (Sigma). Lentivirus genomic RNA was extracted from 125 μl collected supernatants using 375 μl TRIzol-LS reagent and quantified by real-time qPCR. The primers used for qPCR were WPRE-F 5′- GGCACTGACAATTCCGTGGT -3′ and WPRE-R 5′- AGGGACGTAGCAGAAGGACG -3′. The virus titres were calculated according to a standard curve generated by using a series of diluted standard virus samples (Applied Biological Materials Inc.). HEK293 cells were transduced with lentivirus preparations and 8 μg ml^−1^ Hexadimethrine bromide (Sigma) in six-well plates. After 24 h of incubation, the cells received fresh medium containing 1 μg ml^−1^ puromycin (Sigma) and were cultured for an additional 6 days before collection for immunoblotting of the endogenous *P53* gene.

### Small RNA deep sequencing and data analysis

A total of 400 ng plasmids of control, shGP, shGP-LC or saiGP-RZ were transiently transfected into HEK293 cells in a six-well plate for 48 h. Total cellular RNA was extracted to construct a small RNA library according to the Illumina protocol. High-throughput RNA sequencing was performed using Hi-seq2000 with 50 running circles (Genergy Biotechnology). The raw fastq data were pre-processed using a common procedure. After quality filtering, sequencing reads were clipped from the 3′ adaptor and redundant sequences were collapsed as useful reads for further analysis. Reads that could not be clipped or with lengths shorter than 19 bp were removed. The total small RNA reads were obtained by mapping the useful reads to the human genome (version hg19 from UCSC) and shRNA or saiRNA sequences using Bowtie (version 0.12.7) without allowing any mismatches. The mapped genome reads were further categorized as miRNA, tRNA, rRNA and snoRNA sequentially. miRNA annotation was downloaded from miRBase (version 18). Only reads that exactly matched the 5′ start site of annotated mature miRNA and 3′ ends with ≤2 nt deletions or additional sequences derived from primary miRNAs were counted as mature miRNA reads. The remaining reads were mapped against tRNAs, rRNAs and snoRNAs without allowing any mismatches. Annotation of tRNAs was from GtRNAdb, rRNAs from NCBI and Ensembl (NCBI for 18S and 28S, Ensembl for 5S and 5.8S), and snoRNAs from Ensembl. For the correlation assay of miRNA expression in Fig. 5b, the counts for each mature miRNA were normalized to the total miRNA counts and multiplied by 1,000,000.

### Transcriptome sequencing and data analysis

A total of 400 ng of control, shGP, shGP-LC or saiGP-RZ plasmids were transiently transfected into HEK293 cells in six-well plate for 48 h. Total cellular RNA was extracted to construct a complementary DNA library using the NEBNext Ultra Directional RNA Library Prep Kit (NEB) according to the manufacturer's protocol. The complementary DNA libraries were sequenced using Illumina Hi-seq2000 with 2 × 100 running circles. Unique raw R1 fastq reads were mapped to the human transcriptome using Tophat with up to 2 nt mismatches and assembled into transcripts using cufflinks. The human transcriptome annotation was retrieved from Gencode (version 17). The human genome was downloaded from UCSC (version hg19). Only genes with fragments per kb of transcript per million mapped reads (FPKM) ⩾10 were used for further analysis of differential expression. Genes with expression changes of >1.5-fold compared with control samples were considered to be significantly up- or downregulated. For the downregulated genes, the 3′ UTRs of the longest annotated transcripts were searched for sites with reverse complementarity to siRNA seed sequences (nucleotides 2–7 of the most abundant siRNA sequences). For the upregulated genes, 3′ UTRs of the longest annotated transcripts were searched for target sites of the top 50 miRNAs of corresponding samples using TargetScan.

## Additional information

**Accession codes:** The raw fastq data for small RNA-seq and transcriptome RNA-seq were deposited in the GEO database at the National Center for Biotechnology Information under the accession code GSE68631.

**How to cite this article:** Shang, R. *et al*. Ribozyme-enhanced single-stranded Ago2-processed interfering RNA triggers efficient gene silencing with fewer off-target effects. *Nat. Commun.* 6:8430 doi: 10.1038/ncomms9430 (2015).

## Supplementary Material

Supplementary Information Supplementary Figures 1-9 and Supplementary Methods

Supplementary Data 1 Gene expression atlas of the RNA-seq results of the cells transfected with shGP, shGP-LC and saiGP-RZ.

Supplementary Data 2 Predicted off-target sites of down-regulated genes in the cells transfected with shGP, shGP-LC and saiGP-RZ.

Supplementary Data 3 siRNA sequences detected by small RNA-seq in the cells transfected with shRNA, shRNA-LC and saiRNA-RZ targeting EGFP or LaminC.

Supplementary Data 4 Predicted target sites of 50 most abundant miRNAs in up-regulated genes in the cells transfected with shGP, shGP-LC and saiGP-RZ.

Supplementary Data 5 Expression level of different types of small RNAs in the cells transfected with shRNA, shRNA-LC or saiRNA-RZ targeting EGFP or LaminC.

Supplementary Data 6 Expression atlas of endogenous miRNAs detected by small RNA-seq in the cells transfected with shRNA, shRNA-LC and saiRNA-RZ targeting EGFP or LaminC.

## Figures and Tables

**Table 1:** The numbers of up- and downregulated genes in different samples.

	**shGP**	**shGP-LC**	**saiGP-RZ**
Upregulated genes (FC ⩾1.5)	136	93	49
Downregulated genes (FC ⩾1.5)	182	189	101
siGP guide strand seed-dependent (2–7) downregulated genes	103	103	52
siGP passenger strand seed-dependent (2–7) downregulated genes	18	68	NA

FC, fold change; NA, not analysed.
