# Supplementary material for: Ribozyme-enhanced single-stranded Ago2-processed interfering RNA triggers efficient gene silencing with fewer off-target effects
Source: Nat Commun. 2015 Oct 12;6:8430. doi: 10.1038/ncomms9430 (PMC4633630; doi:10.1038/ncomms9430)
Supplement: Supplementary Information — Supplementary Figures 1-9 and Supplementary Methods [file ncomms9430-s1.pdf]

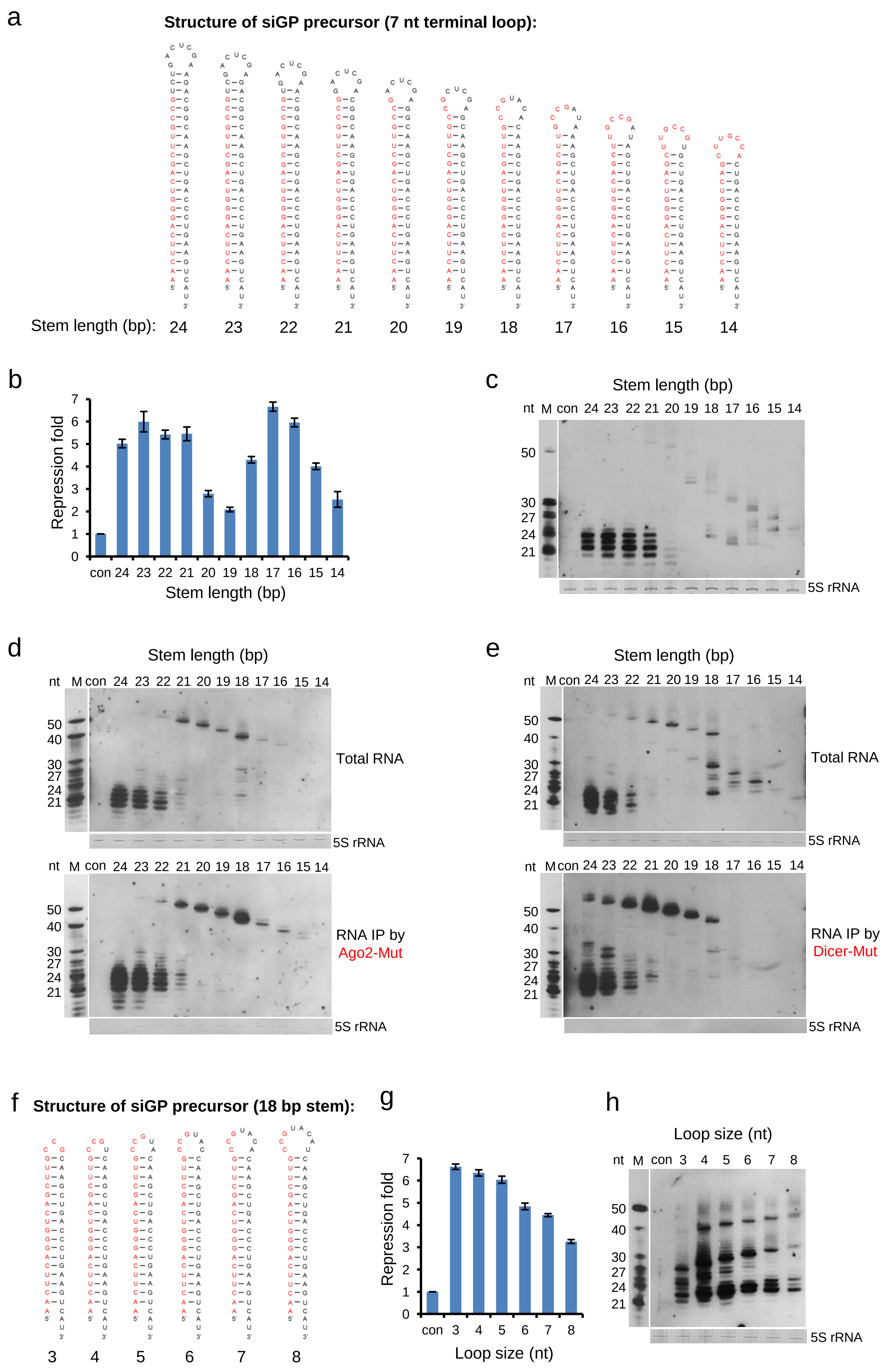

Supplementary Figure 1. Processing and repression efficiency of shRNAs with different stem lengths or loop sizes. (**a**) Structure of shGP with a 7 nt top loop and stems ranging from 14 to 24 bp. (**b**) Knockdown efficiency of the shRNAs in (**a**) measured by luciferase assays. (**c**) Processing of the shRNAs in (**a**) detected by Northern blotting. (**d**) Association of Ago2 with shRNAs of different stem length. shGP described in Fig. 1a were cotransfected into HEK293 cells with N-terminally HA-tagged Ago2-Mut (D597A) deprived of cleavage activity. Forty-eight hours after transfection, HA-Ago2-Mut was immunoprecipitated using an HA-specific antibody. The total RNA and IP RNA were detected using a siGP-specific probe in Northern blotting assay. (**e**) Association of Dicer with shRNAs of different stem length. shGP described in Fig. 1a were cotransfected into HEK293 cells with N-terminally myc-tagged Dicer-Mut (D1320A, D1709A) deprived of cleavage activity. Forty-eight hours after transfection, myc-Dicer-Mut was immunoprecipitated by myc specific antibody, after which the total RNA and IP RNA were detected by siGP-specific probe in Northern blotting. (**f**) Structure of shGP with a 18 bp stem and top loops ranging from 3 to 8 nt. (**g**) Knockdown efficiency of the shRNAs in (**f**) measured by luciferase assays. (**h**) Processing of the shRNAs in (**f**) detected by Northern blotting. All the error bars represent the standard deviation of three independent measurements.

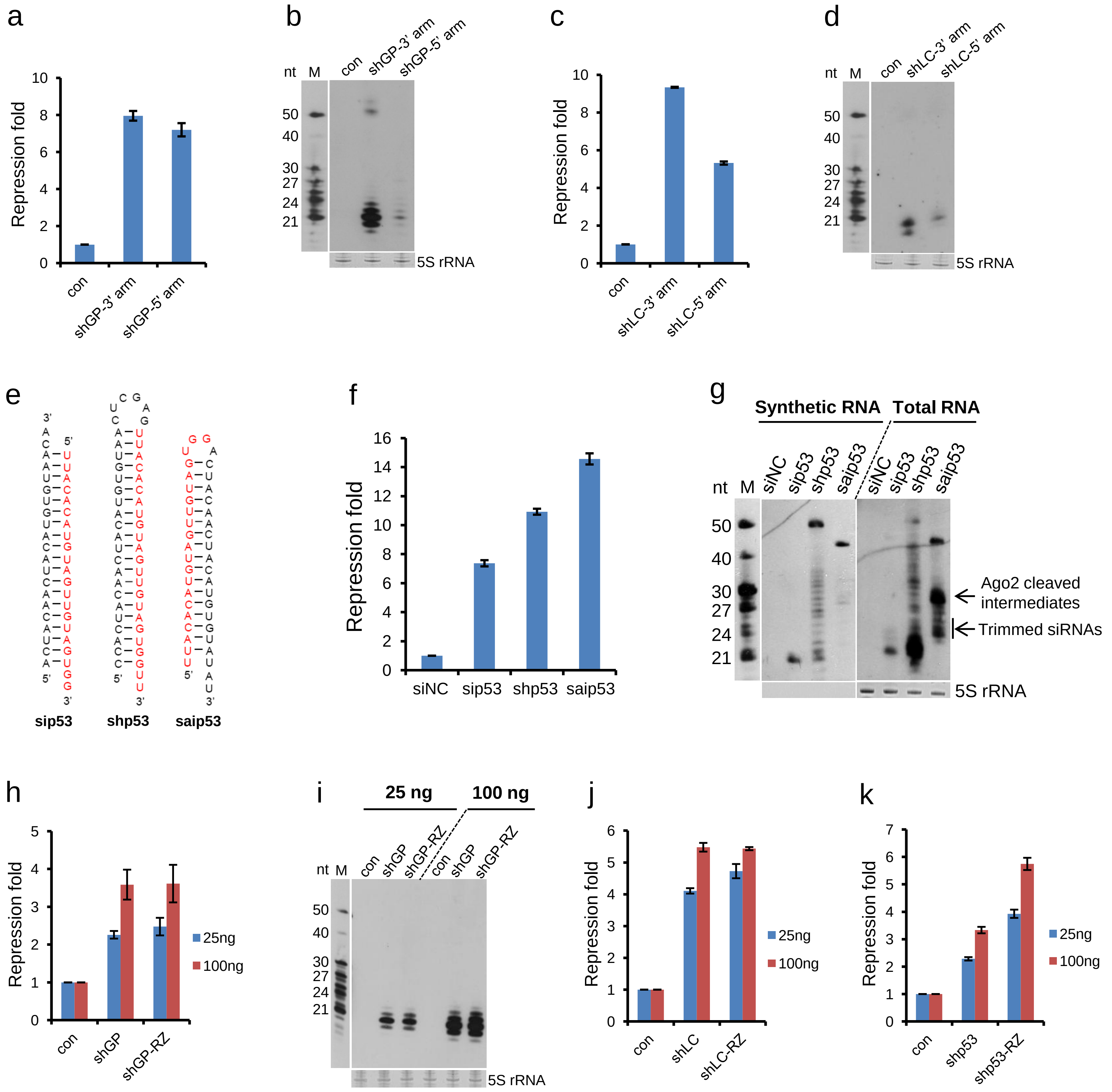

Supplementary Figure 2. Effect of guide strand location in shRNA and comparison of H1 promoter transcribed or chemically synthesized shRNA and saiRNA. (**a**, **c**) Knockdown efficiency of siGP or siLC located on the 5' or 3' strand of the shRNAs using a luciferase assay as in Fig. 2d. (**b**, **d**) Processing of siGP or siLC located on the 5' or 3' strand of the shRNAs detected by Northern blotting as in Fig. 2d. (**e**) Structures of chemically synthesized sip53, shp53 and saip53. The guide strand is labeled in red. (**f**) Knockdown efficiency of different siRNA precursors in (**e**) measured using a luciferase assay as in Fig. 1j. (**g**) Processing of different siRNA precursors in (**e**) detected by Northern blotting as in Fig. 1k. (**h**) Repression efficiency of shGP and shGP-RZ with different transfection dosages measured by luciferase assay in HEK293 cells. (**i**) Northern blotting of shGP and shGP-RZ expression with different transfection dosages as in (**h**). (**j, k**) Repression efficiency of shRNA and shRNA-RZ targeting *laminC* (**j**) or *P53* genes (**k**) with different transfection dosages measured by luciferase assay in HEK293 cells. All the error bars represent the standard deviation of three independent measurements.

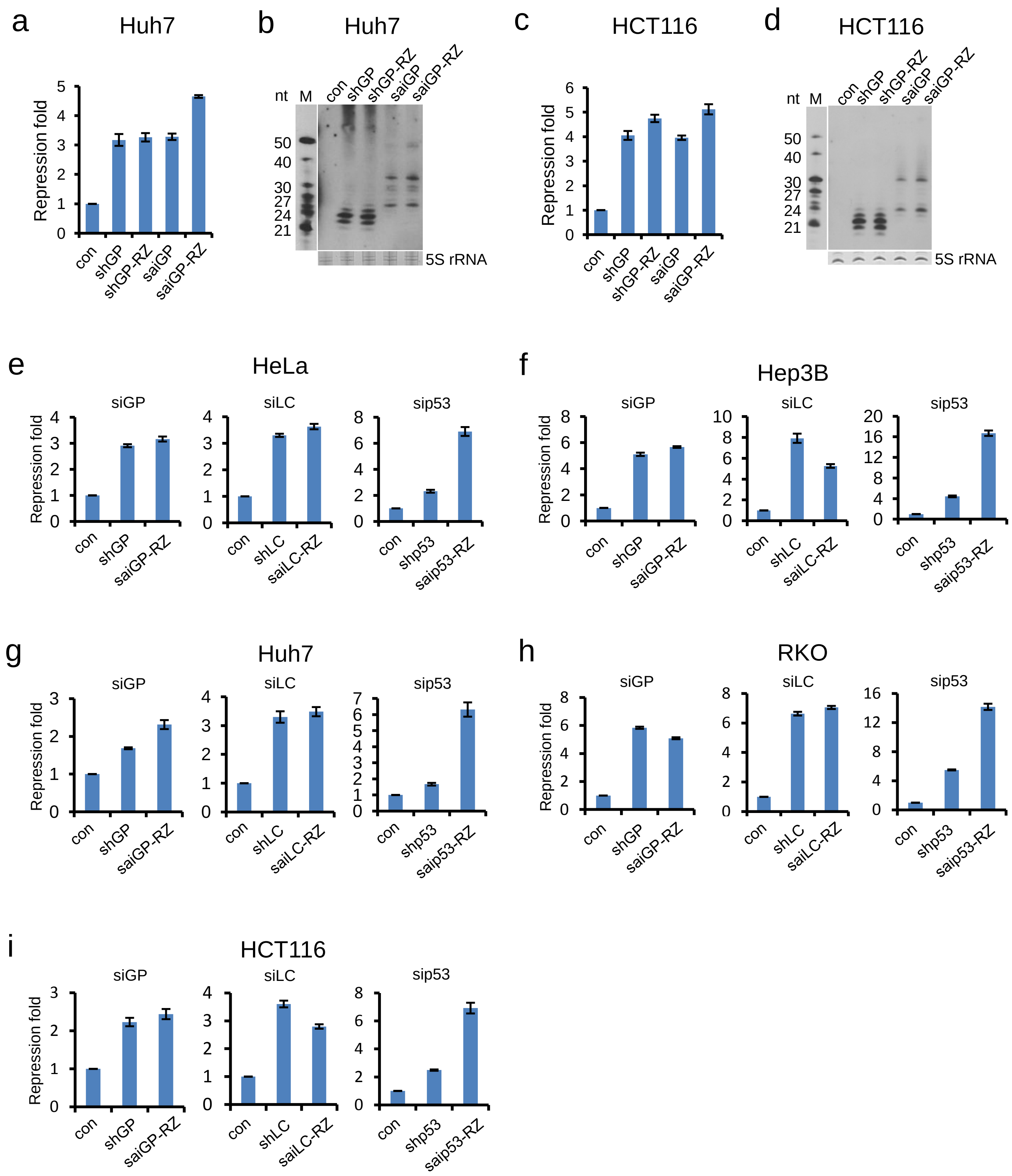

Supplementary Figure 3. Knockdown efficiency and processing of shRNAs and saiRNAs in different cell lines. (**a-d**) Knockdown efficiency and processing of shRNA, shRNA-RZ, saiRNA and saiRNA-RZ targeting the *EGFP* gene in Huh7 (**a**, **b**) and HCT116 (**c**, **d**) cells detected by luciferase assay and Northern blotting as in Fig. 2d. (**e-i**) Knockdown efficiency of shRNA and saiRNA-RZ targeting the *EGFP*, *laminC* and *P53* genes in HeLa (**e**), Hep3B (**f**), Huh7 (**g**), RKO (**h**) and HCT116 (**i**) cells measured using a luciferase assay as in Fig. 2d. All the error bars represent the standard deviation of three independent measurements.

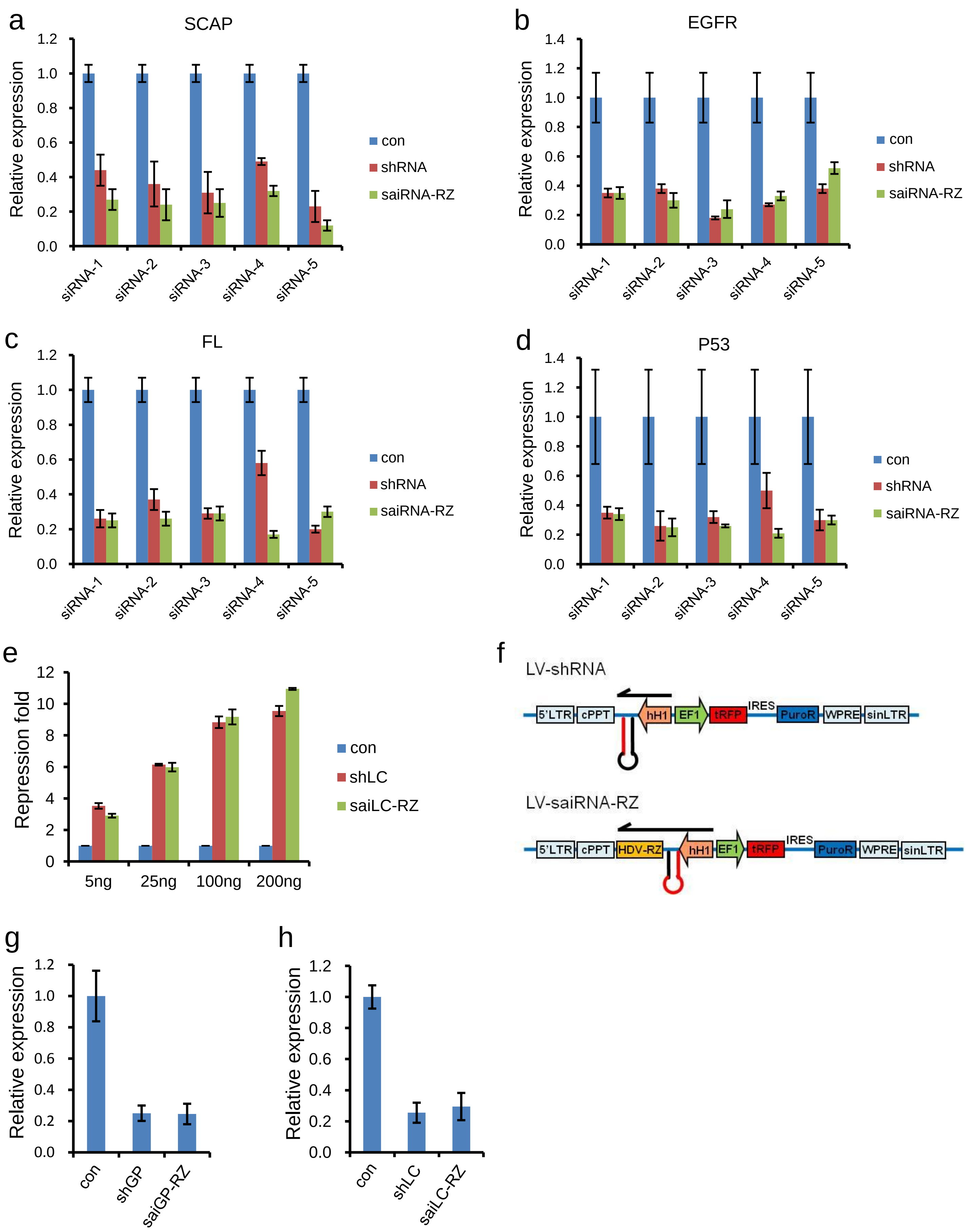

Supplementary Figure 4. Comparison of knockdown efficiency by shRNAs and saiRNA-RZs and schematic diagram of lentiviral vector for expressing saiRNA-RZ. (**a**-**d**) Knockdown efficiency of shRNAs and saiRNA-RZs targeting the *SCAP* (**a**), *EGFR* (**b**), *FL* (**c**) and *P53* (**d**) genes in HEK293 cells measured by RT-qPCR. HEK293 cells were cotransfected with shRNA, saiRNA-RZ or an empty plasmid and a plasmid overexpressing *SCAP*, *EGFR* or *FL*. The abundances of *SCAP*, *EGFR*, *FL* and endogenous *P53* mRNAs were detected by RT-qPCR with gene-specific primers, and the knockdown efficiency was calculated in the presence versus the absence of each shRNA. (**e**) Effect of transfection dosages on the knockdown efficiency of shLC and saiLC-RZ measured using a luciferase assay in HEK293 cells as in Fig. 2g. (**f**) Schematic representations of lentiviral vector encoding shRNA or saiRNA-RZ. The cassette containing H1 and shRNA/saiRNA-RZ was placed in reverse orientation relative to the genomic sequence of lentivirus. (**g**, **h**) Knockdown efficiency of lentiviral shRNA and saiRNA-RZ targeting the *EGFP* (**g**) or *laminC* (**h**) genes in HEK293 cells stably expressing *EGFP* (HEK293-EGFP). The abundance of endogenous *EGFP* or *laminC* genes in HEK293-EGFP cells transduced with lentiviral shRNA, saiRNA-RZ or an empty vector was detected by qPCR with gene-specific primers. The knockdown efficiency of each gene was calculated in the presence versus the absence of each shRNA. β-actin mRNA was used as an internal standard. All the error bars represent the standard deviation of three independent measurements.

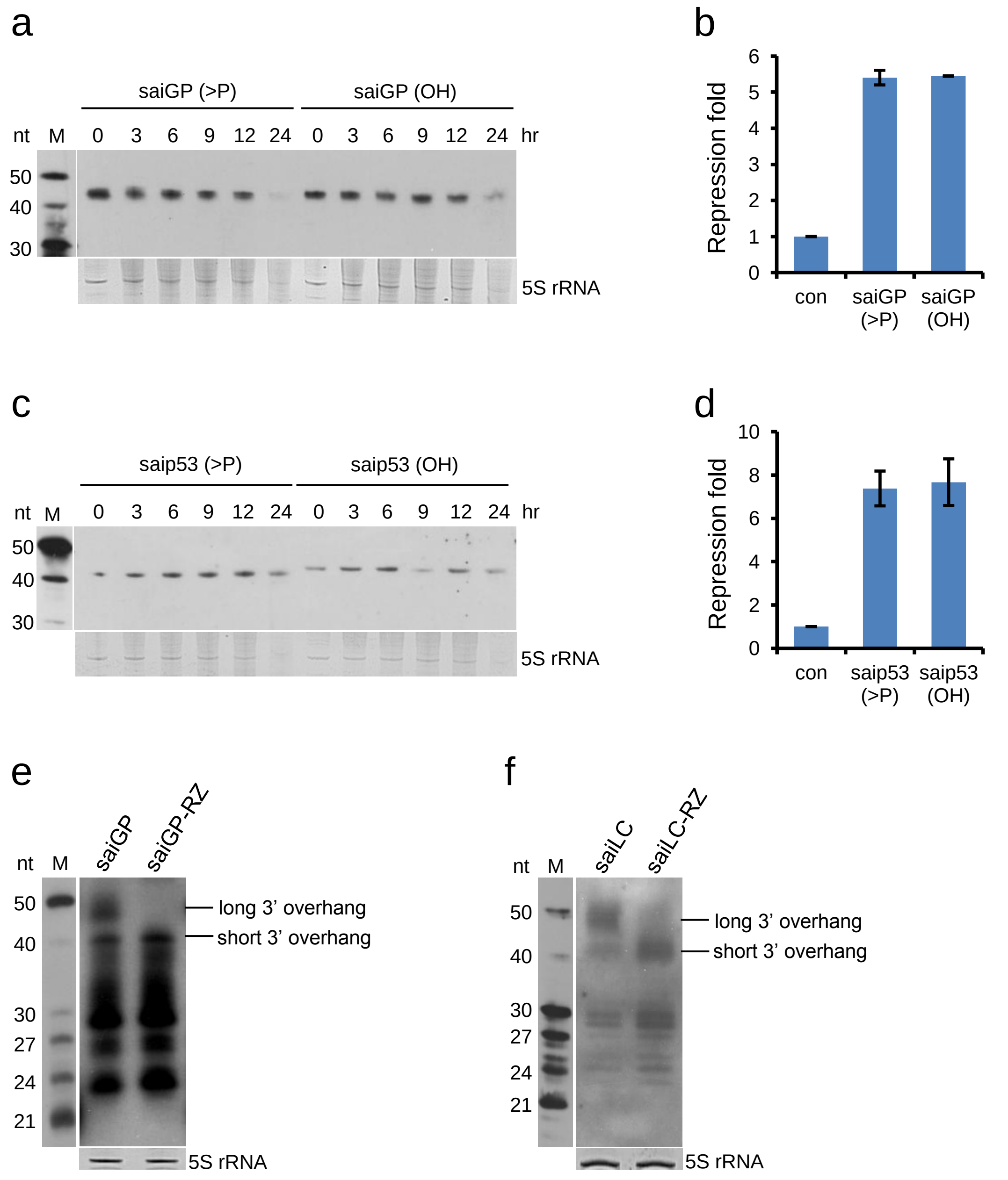

Supplementary Figure 5. The influence on saiRNA precursors caused by 3' overhang length and terminal 2', 3'- cyclic phosphate. (**a, c**) Influence on the stability of saiRNA precursors due to terminal 2', 3'-cyclic phosphate. T7-transcribed saiGP-RZ (**a**) or saip53-RZ (**c**) (as in Fig. 2b) was treated with or without T4 PNK and then incubated with Ago2-KO 293 cell lysate for different time periods. RNA was then extracted for Northern blotting analysis. (**b, d**) Influence on the repression efficiency of saiRNA precursors due to terminal 2', 3'-cyclic phosphate. T7-transcribed saiRNA precursors with or without T4 PNK-treatment were cotransfected into HEK293 cells with a FL reporter containing a siGP (**b**) or sip53 (**d**) target in its 3' UTR and a non-target RL reporter. The expression of the luciferase gene was measured and analyzed as in Fig. 1b. The error bars represent the standard deviation of three independent measurements. (**e, f**) The two isoforms of saiGP (**e**) and saiLC (**f**) precursors detected by Northern blotting.

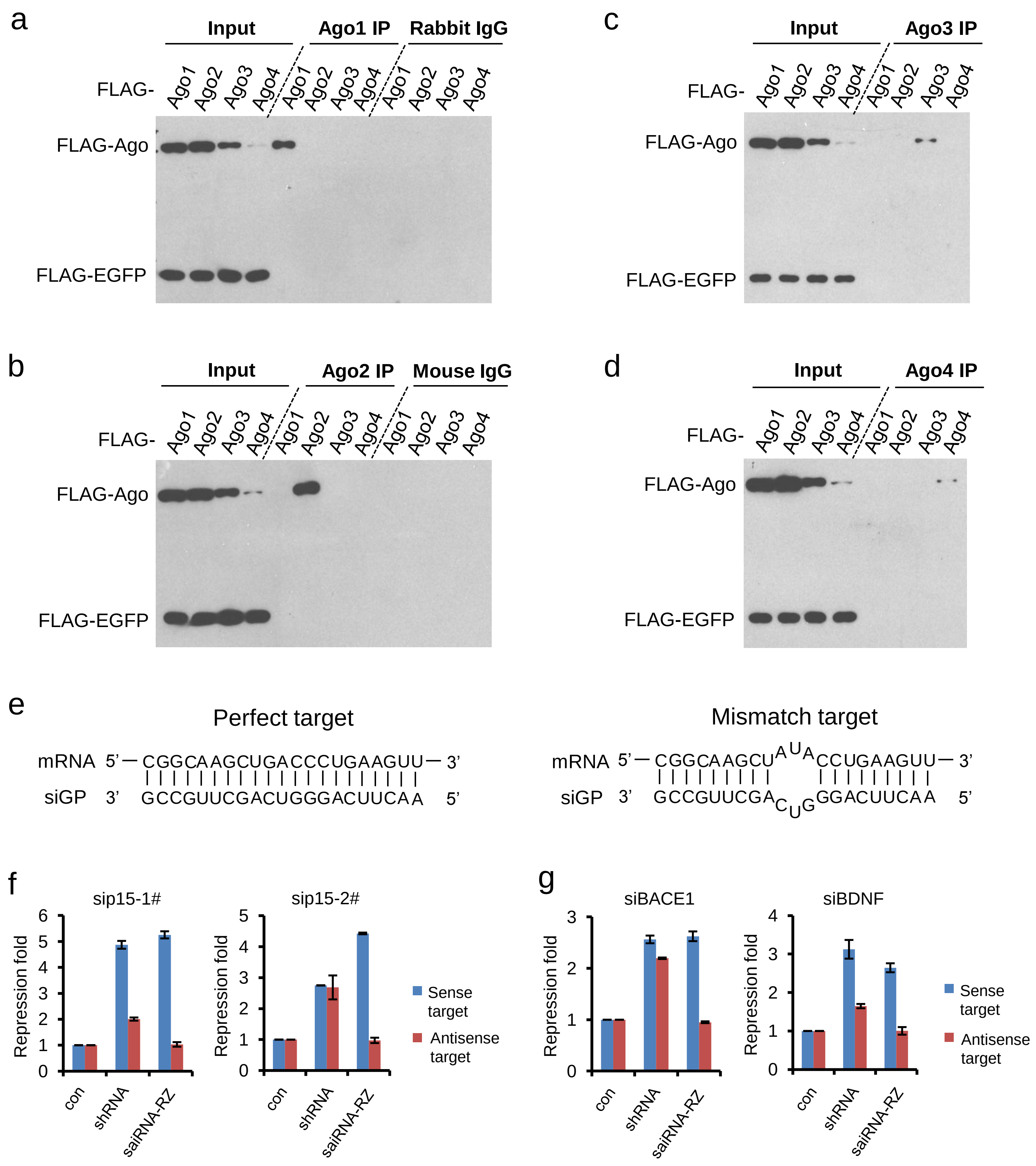

Supplementary Figure 6. Specificity of Ago antibodies and repression of antisense transcripts by the passenger strand of shRNAs. (**a-d**) FLAG-tagged Ago1, 2, 3 or 4 were ectopically expressed in HEK293 cells; immunoprecipitated by Ago1, 2, 3 or 4 specific antibodies; and immunoblotted using an anti-FLAG antibody. A plasmid encoding FLAG-EGFP was cotransfected with the FLAG-Ago expression plasmids and served as an internal control. (**e**) The structure of on-target (perfect target) and off-target (mismatch target) sequences base-pairing with siGP used for the reporter assay in Fig. 4e,f. (**f**) Repression of luciferase reporters bearing the overlap region of sense and antisense transcripts of the *p15* gene by shp15 or saip15-RZ. The guide strand of shp15 or saip15-RZ was designed to target the sense transcripts of the *p15* gene. The plasmid encoding shRNA or saiRNA-RZ was cotransfected into HEK293 cells with a firefly luciferase reporter bearing the overlap region of the sense and antisense transcripts of the *p15* gene in the 3' UTR in the forward (sense target) or reverse (antisense target) orientations. Repression fold was calculated as in Fig. 1b. (**g**) Repression of reporters bearing the overlap region of the sense and antisense transcripts of the *BACE1* and *BDNF* genes by shRNA or saiRNA-RZ. All the error bars represent the standard deviation of three independent measurements.

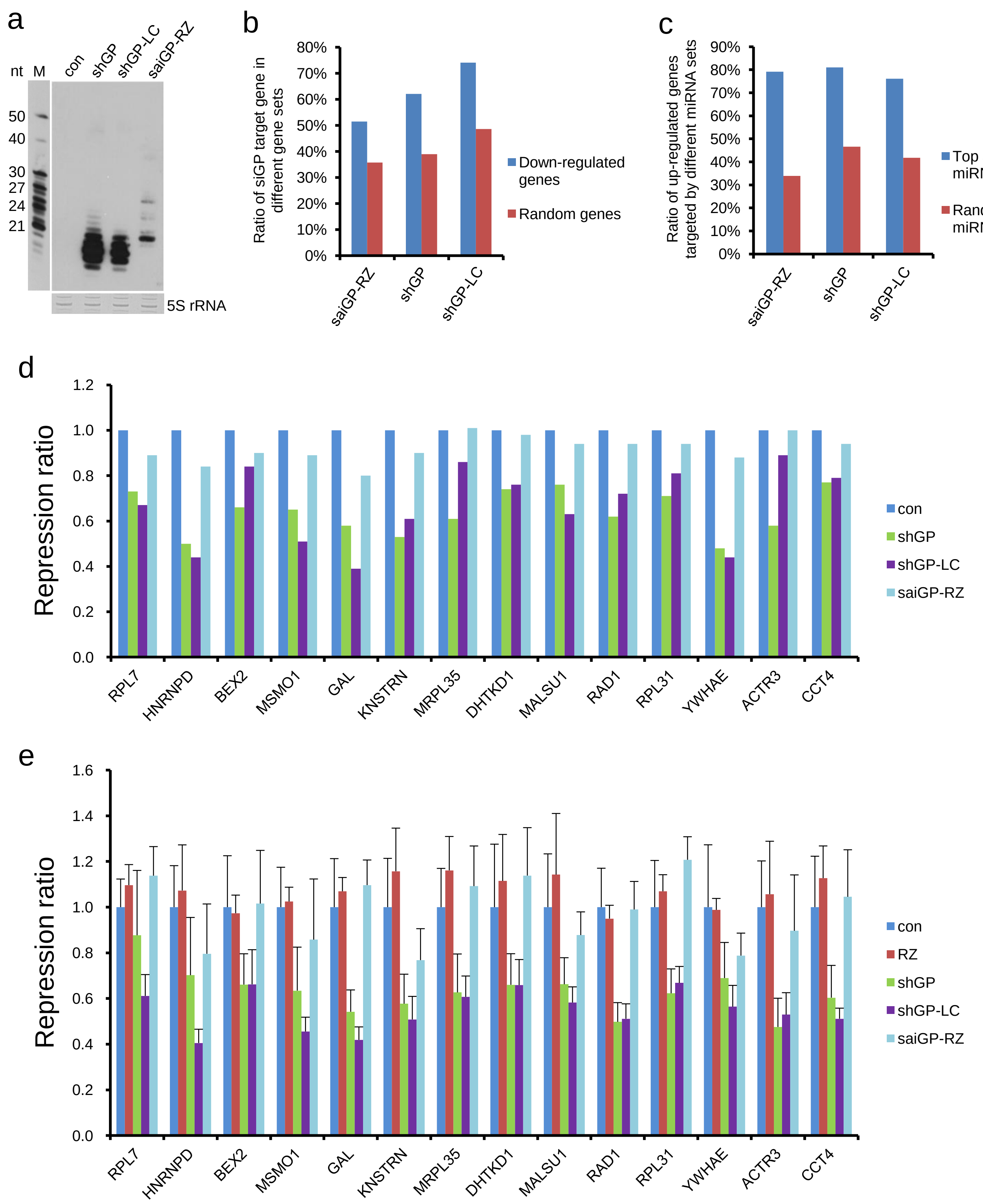

Supplementary Figure 7. saiRNA produces reduced off-target effects on endogenous genes than shRNA. (**a**) Northern blotting of siRNAs in HEK293 cells transfected with shGP, shGP-LC and saiGP-RZ as used for transcriptome analysis and small RNA sequencing. (**b**) Correlation between down-regulated genes and seed sequences of siGP in HEK293 cells. The ratio of genes containing sequences complementary to the siGP seed region (2-7) in the 3' UTR of down-regulated (FC ≥1.5) or random genes was calculated for samples transfected with saiGP-RZ, shGP or shGP-LC. (**c**) Correlation between up-regulated genes and the seed sequences of the 50 most abundant miRNAs in HEK293 cells. The ratio of up-regulated (FC ≥1.5) genes containing at least one site complementary to a seed region (2-7) of one of the top 50 or random 50 miRNAs in the 3' UTR was calculated for samples transfected with saiGP-RZ, shGP or shGP-LC. (**d**) The relative expression level of 14 randomly selected genes with significant off-target effects detected from the transcriptome analysis in Fig. 4g. (**e**) The expression level of the 14 genes in (**d**) as measured by quantitative RT-PCR. The error bars represent the standard deviation of three independent measurements.

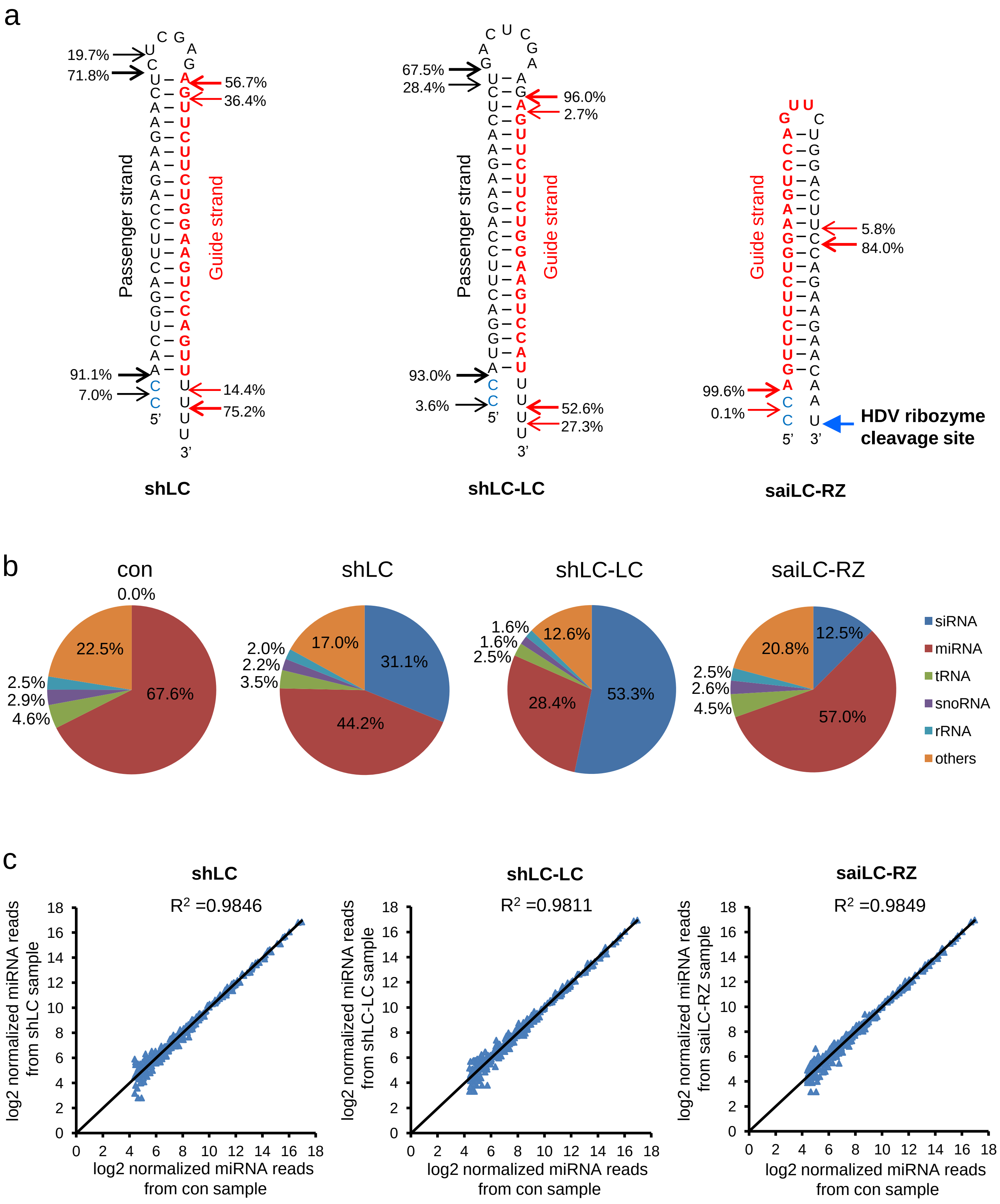

Supplementary Figure 8. saiRNA exhibits less competition with endogenous miRNA than shRNA. (**a**) Processing of shLC, shLC-LC and saiLC-RZ analyzed by deep sequencing. Small RNAs from HEK293 cells transfected with plasmids encoding shLC, shLC-LC or saiLC-RZ were subjected to deep-sequencing analysis. The 5' and 3' end positions of the two most abundant isoforms of the guide strand or passenger strand are labeled with red or black arrows, respectively, with the relative abundance indicated next to the arrows. The nucleotides in blue indicate the sequence of H1 promoter. The blue arrow indicates the cleavage site of HDV ribozyme. (**b**) Relative abundance of endogenous small non-coding RNAs and siRNAs in HEK293 cells transfected with shLC, shLC-LC, saiLC-RZ or an empty vector (con) measured by deep sequencing. (**c**) Correlations of the 300 most abundant miRNAs in cells transfected with shLC, shLC-LC, saiLC-RZ or an empty vector. Expression levels of individual miRNA were normalized to total miRNA counts in each sample and the top 300 miRNAs were used for correlation analysis between control and shLC, shLC-LC or saiLC-RZ.

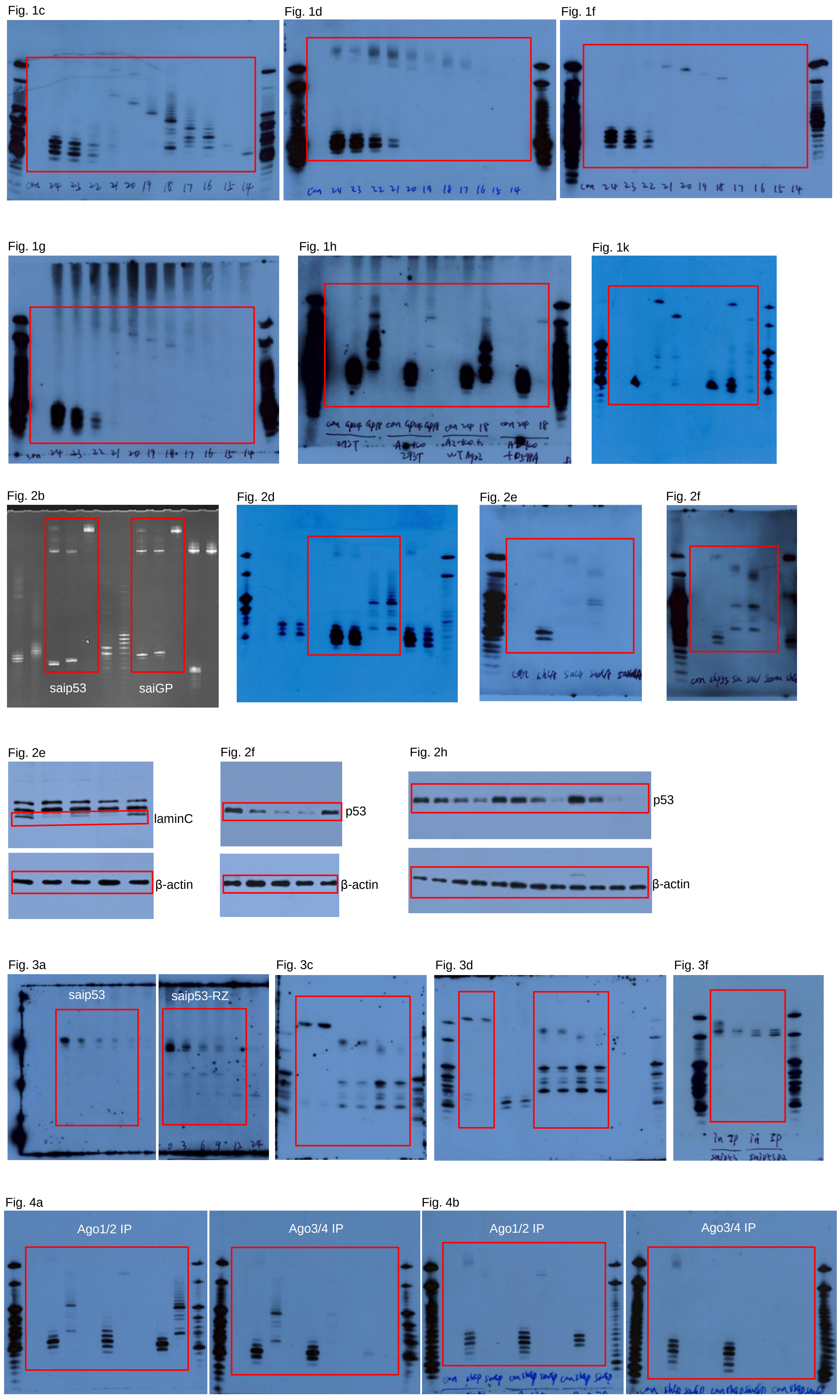

Supplementary Figure 9. Uncropped scans of Northern and Western blots.

Fig. 4c
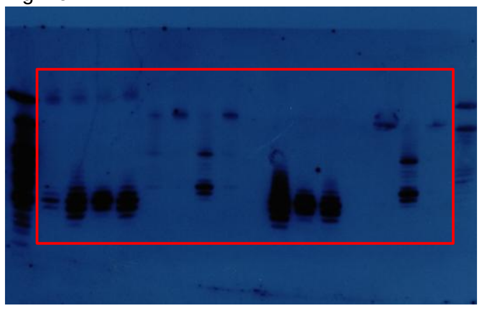

Fig. 4d
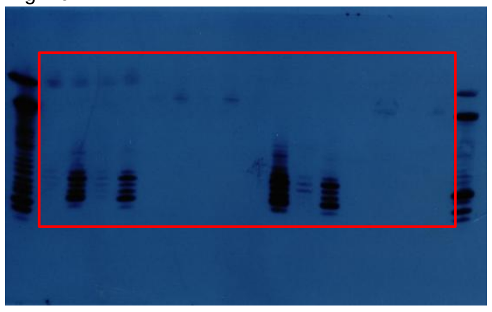

Fig. S1c
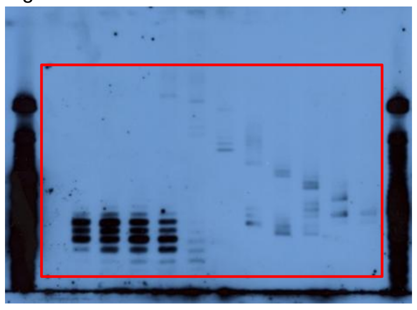

Fig. S1f
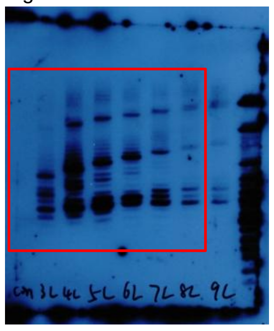

Fig. S1g
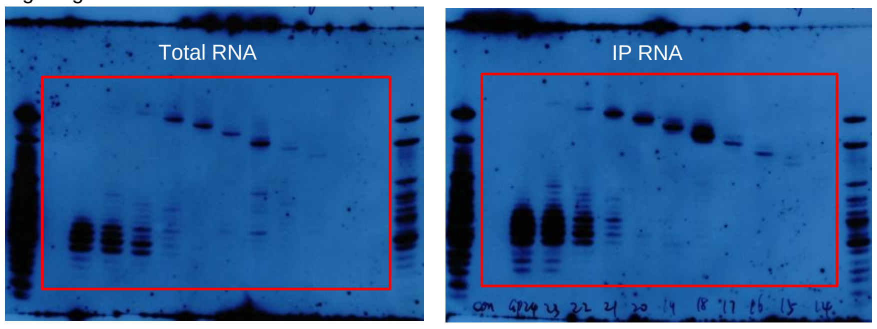

Fig. S1h
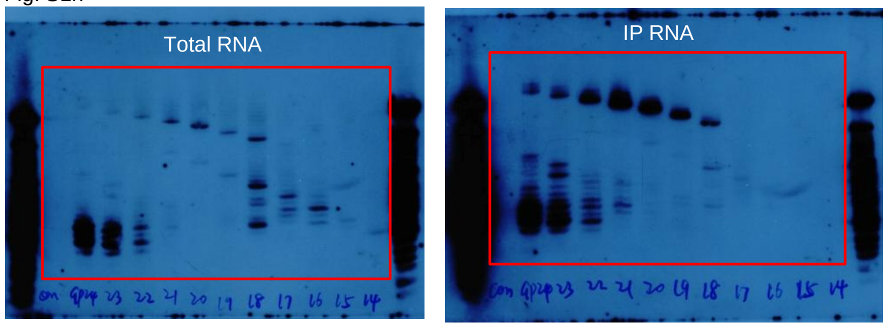

Fig. S2b
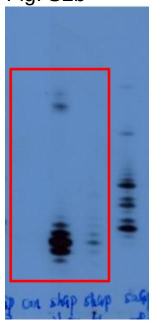

Fig. S2d
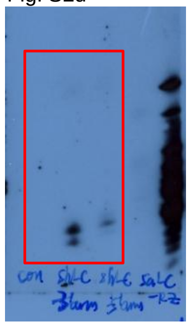

Fig. S2g
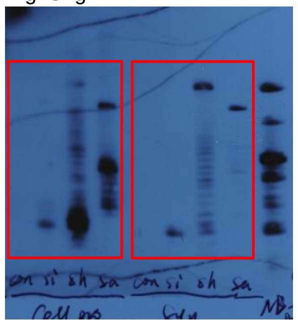

Fig. S2i
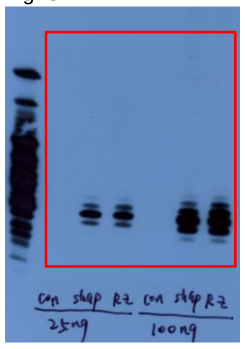

Fig. S3b
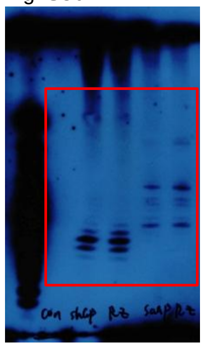

Fig. S3d
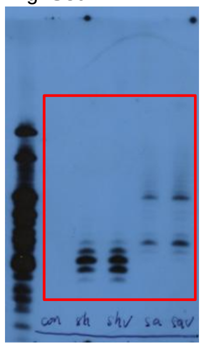

Fig. S5a
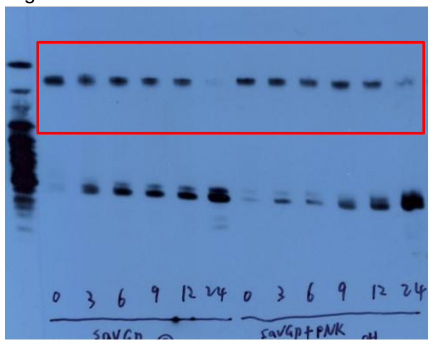

Fig. S5c
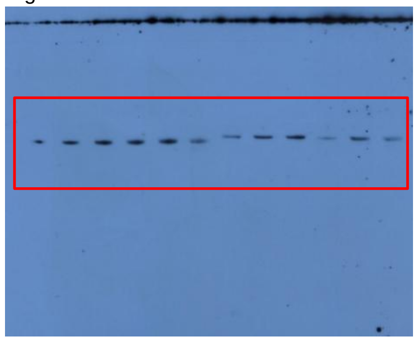

Fig. S5e
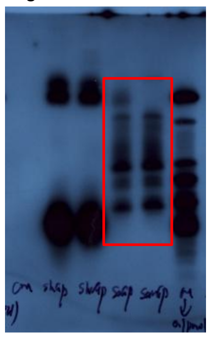

Fig. S5f
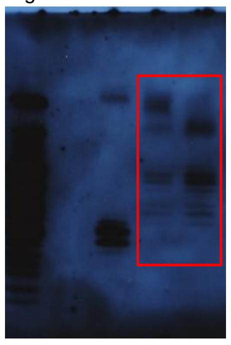

Fig. S6a, b
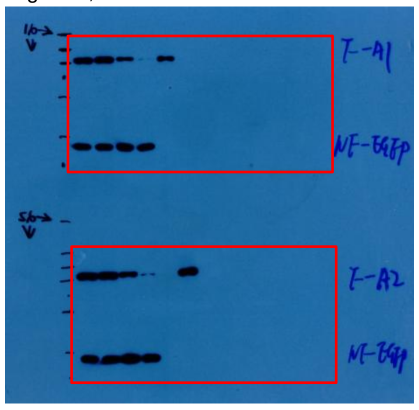

Fig. S6c, d
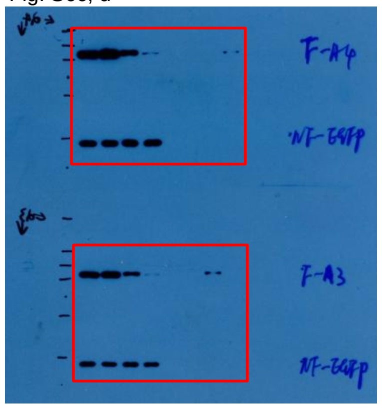

Fig. S7a
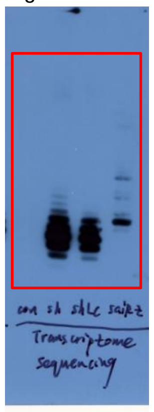

Supplementary Figure 9 continued. Uncropped scans of Northern and Western blots.

Supplementary Methods

DNA oligonucleotides for shRNA and saiRNA plasmids:

shGP-F:
GATCCCGGCAAGCTGACCCTGAAGTTCTCGAGAACTTCAGGGTCAGCTTGCCGTTTTTGGAAA
shGP-R:
AGCTTTTCCAAAAACGGCAAGCTGACCCTGAAGTTCTCGAGAACTTCAGGGTCAGCTTGCCGG

shGP-5’arm-F:
GATCCAACTTCAGGGTCAGCTTGCCGCTCGAGCGGCAAGCTGACCCTGAAGTTTTTTTGGAAA
shGP-5’arm-R:
AGCTTTTCCAAAAAAACTTCAGGGTCAGCTTGCCGCTCGAGCGGCAAGCTGACCCTGAAGTTG

shGP-LC-F:
GATCCGCAAGCTGACCCTGAAGTTCTGACTCGAAGAACTTCAGGGTCAGCTTGCTTTTTGGAAA
shGP-LC-R:
AGCTTTTCCAAAAAGCAAGCTGACCCTGAAGTTCTTCGAGTCAGAACTTCAGGGTCAGCTTGCG

saiGP-F:
GATCCAACTTCAGGGTCAGCTTGCCGTCAAGCTGACCCTGAAGTCATTTTTTGGAAA
saiGP-R:
AGCTTTTCCAAAAAATGACTTCAGGGTCAGCTTGACGGCAAGCTGACCCTGAAGTTG

shp53-F:
GATCCCCACTACAACTACATGTGTATCTCGAGATACACATGTAGTTGTAGTGGTTTTTGGAAA
shp53-R:
AGCTTTTCCAAAAACCACTACAACTACATGTGTATCTCGAGATACACATGTAGTTGTAGTGGG

saip53-F:
GATCCATACACATGTAGTTGTAGTGGACTACAACTACATGTGTAAATTTTTTGGAAA
saip53-R:
AGCTTTTCCAAAAAATTTACACATGTAGTTGTAGTCCACTACAACTACATGTGTATG

shLC-F:
GATCCAACTGGACTTCCAGAAGAACTCTCGAGAGTTCTTCTGGAAGTCCAGTTTTTTTGGAAA

shLC-R:
AGCTTTTCCAAAAAAACTGGACTTCCAGAAGAACTCTCGAGAGTTCTTCTGGAAGTC
CAGTTG

shLC-5’arm-F:
GATCCAGTTCTTCTGGAAGTCCAGTTCTCGAGAACTGGACTTCCAGAAGAACTTTTT
TGGAAA
shLC-5’arm-R:
AGCTTTTCCAAAAAAGTTCTTCTGGAAGTCCAGTTCTCGAGAACTGGACTTCCAGAA
GAACTG

shLC-LC-F:
GATCCATGGACTTCCAGAAGAACTCTGACTCGAAGAGTTCTTCTGGAAGTCCATTTTT
TGGAAA
shLC-LC-R:
AGCTTTTCCAAAAAATGGACTTCCAGAAGAACTCTTCGAGTCAGAGTTCTTCTGGAA
GTCCATG

saiLC-F:
GATCCAGTTCTTCTGGAAGTCCAGTTCTGGACTTCCAGAAGAACAATTTTTTGGAAA
saiLC-R:
AGCTTTTCCAAAAAATTGTTCTTCTGGAAGTCCAGAACTGGACTTCCAGAAGAACTG

shGP-RZ-F:
ACGTGGATCCCGGCAAGCTGACCCTGAAGTTCTCGAGAACTTCAGGGTCAGCTTGC
CGATGGCCGGCATGGTCCCAGC
saiGP-RZ-F:
ACGTGGATCCAACTTCAGGGTCAGCTTGCCGTCAAGCTGACCCTGAAGTCATGGCC
GGCATGGTCCCAGCCT

shLC-RZ-F:
ACGTGGATCCAACTGGACTTCCAGAAGAACTCTCGAGAGTTCTTCTGGAAGTCCAGT
TATGGCCGGCATGGTCCCAGC
saiLC-RZ-F:
ACGTGGATCCAGTTCTTCTGGAAGTCCAGTTCTGGACTTCCAGAAGAACAATGGCCG
GCATGGTCCCAGCCT

shp53-RZ-F:
ACGTGGATCCCCACTACAACTACATGTGTATCTCGAGATACACATGTAGTTGTAGTGG
ATGGCCGGCATGGTCCCAGC
saip53-RZ-F:
ACGTGGATCCATACACATGTAGTTGTAGTGGACTACAACTACATGTGTAAATGGCCG
GCATGGTCCCAGCCT
HDVRZ-R:

ATCTAAGCTTTTCCAAAAAAGTGGGTCCCATTCGCCA

miR-p53-F:
TCGAGGTTACACATGTAGTTGTAGTGGAGTGTGCTGTCCTCCACTACAACTACATGTG
TATCCTCGCA
miR-p53-R:
CCGGTGCGAGGATACACATGTAGTTGTAGTGGAGGACAGCACACTCCACTACAACTA
CATGTGTAACC

shp15-1F:
CTAGATTCCAAAAAGAGAGCAATTGTAACGGTTATCTCGAGATAACCGTTACAATTGCT
CTCG
shp15-1R:
GATCCGAGAGCAATTGTAACGGTTATCTCGAGATAACCGTTACAATTGCTCTCTTTTT
GGAAT

saip15-RZ-1F:
CCGGCCATTTAACCGTTACAATTGCTTGAGAGCAATTGTAACGGTTATG
saip15-RZ-1R:
GATCCATAACCGTTACAATTGCTCTCAAGCAATTGTAACGGTTAAATGGCCGGCATG

shp15-2F:
CTAGATTCCAAAAAAGGGAAGAGTGTCGTTAAGTTCTCGAGAACTTAACGACACTCTT
CCCTG
shp15-2R:
GATCCAGGGAAGAGTGTCGTTAAGTTCTCGAGAACTTAACGACACTCTTCCCTTTTTT
GGAAT

saip15-RZ-2F:
CCGGCCATTACTTAACGACACTCTTCAAGGGAAGAGTGTCGTTAAGTTG
saip15-RZ-2R:
GATCCAACTTAACGACACTCTTCCCTTGAAGAGTGTCGTTAAGTAATGGCCGGCATG

shBACE1-F:
CTAGATTCCAAAAAGACAAGAGCATTGTGGACAGTCTCGAGACTGTCCACAATGCTC
TTGTCG
shBACE1-R:
GATCCGACAAGAGCATTGTGGACAGTCTCGAGACTGTCCACAATGCTCTTGTCTTTT
TGGAAT

saiBACE1-RZ-F:
CCGGCCATTCTGTCCACAATGCTCTTTGACAAGAGCATTGTGGACAGTG
saiBACE1-RZ-R:
GATCCACTGTCCACAATGCTCTTGTCAAAGAGCATTGTGGACAGAATGGCCGGCATG

shBDNF-F:
CTAGATTCCAAAAACAGTAGTCAAGTGCCTTTGGTCTCGAGACCAAAGGCACTTGAC
TACTGG
shBDNF-R:
GATCCCAGTAGTCAAGTGCCTTTGGTCTCGAGACCAAAGGCACTTGACTACTGTTTT
TGGAAT

saiBDNF-RZ-F:
CCGGCCATTCCAAAGGCACTTGACTATCAGTAGTCAAGTGCCTTTGGTG
saiBDNF-RZ-R:
GATCCACCAAAGGCACTTGACTACTGATAGTCAAGTGCCTTTGGAATGGCCGGCATG

siRNA sequence selected for shRNA and saiRNA comparison:
siSCAP-1: AGAAGATGGTTGCAATGTGATG
siSCAP-2: ATGATGTTGTAGTAGTTGAAGA
siSCAP-3: AAGTTGGTGTCGATTAAGCAGG
siSCAP-4: AATCAAGGGAACCATTAAGCCG
siSCAP-5: AAAGTAGACTGTCTGTAACAGG
siEGFR-1: ATTGGAGTCTGTAGGACTTGGC
siEGFR-2: AATTCGATGATCAACTCACGGA
siEGFR-3: ATGTAGACATCGATGGTACATA
siEGFR-4: ATGAAATTATCACATCTCCATC
siEGFR-5: AGAGGAGTATGTGTGAAGGAGT
siFL-1: AAAGTACTCAGCGTAAGTGATG
siFL-2: AGTTGGAGCAAGATGGATTCCA
siFL-3: AAAGAAGTGTTCGTCTTCGTCC
siFL-4: AAACCGGACATAATCATAGGAC
siFL-5: AGGGTGTAATCAGAATAGCTGA
sip53-1: AAATCATCCATTGCTTGGGACG
sip53-2: AGTCATCCAAATACTCCACACG
sip53-3: ACCAGTGGTTTCTTCTTTGGCT
sip53-4: AGAAGGGTGAAATATTCTCCAT
sip53-5: AACACATGTAGTTGTAGTGGAT

DNA oligonucleotides for target reporter plasmids:
2×GPM-F:
CTAGCTCCGGCAAGCTGATACGCCCTGAAGTTCAATCCGGCAAGCTGATACGCCCT
GAAGTTCAAT
2×GPM-R:
CTAGATTGAACTTCAGGGCGTATCAGCTTGCCGGATTGAACTTCAGGGCGTATCAG
CTTGCCGGAG

2×LCM-F:

CTAGCTCAACTGGACTTCGCTGCAGAAGAACTCAATCAACTGGACTTCGCTGCAGAAGAACTCAAT
2×LCM-R:
CTAGATTGAGTTCTTCTGCAGCGAAGTCCAGTTGATTGAGTTCTTCTGCAGCGAAGTCCAGTTGAG

2×P53M-F:
CTAGCTCCCACTACATGAACATGTGTATCAATCCCACTACATGAACATGTGTATCAAT
2×P53M-R:
CTAGATTGATACACATGTTCATGTAGTGGGATTGATACACATGTTCATGTAGTGGGAG

2×GPSM-F:
CTAGCGAGAACTTCAGCCACAGCTTGCCGGGAGAGAACTTCAGCCACAGCTTGCCGGGAT
2×GPSM-R:
CTAGATCCCGGCAAGCTGTGGCTGAAGTTCTCTCCCGGCAAGCTGTGGCTGAAGTTCTCG

2×LCSM-F:
CTAGCGAGAGTTCTTCACCAAGTCCAGTTGGAGAGAGTTCTTCACCAAGTCCAGTTGGAT
2×LCSM-R:
CTAGATCCAACTGGACTTGGTGAAGAACTCTCTCCAACTGGACTTGGTGAAGAACTCTCG

2×P53SM-F:
CTAGCGAGATACACATCATGTTGTAGTGGGGAGAGATACACATCATGTTGTAGTGGGGAT
2×P53SM-R:
CTAGATCCCCACTACAACATGATGTGTATCTCTCCCCACTACAACATGATGTGTATCTCG

P53P-F: TCGAGCACTCCACTACAACTACATGTGTAACCTT
P53P-R: CTAGAAGGTTACACATGTAGTTGTAGTGGAGTGC

LCP-F: CTAGCACACAAACTGGACTTCCAGAAGAACACTGT
LCP-R: CTAGACAGTGTTCTTCTGGAAGTCCAGTTTGTGTG

GPSP-F: TCGAGGAGAACTTCAGGGTCAGCTTGCCGGGAT
GPSP-R: CTAGATCCCGGCAAGCTGACCCTGAAGTTCTCC

P53SP-F: TCGAGGAGATACACATGTAGTTGTAGTGGGGAT
P53SP-R: CTAGATCCCCACTACAACTACATGTGTATCTCC

LCSP-F: TCGAGGAGAGTTCTTCTGGAAGTCCAGTTGGAT
LCSP-R: CTAGATCCAACTGGACTTCCAGAAGAACTCTCC

p15-F: ATCGGCTAGCGGGTAATGAAGCTGAGCCCAGGTCT
p15-R: ATCGTCTAGAGTAGGACATCCCACGAGCCATCATAT

BDNF-F: GATCGCTAGCTACCCAGGTGTGCGGACCCAT
BDNF-R: GATCTCTAGACAGCAGAAAGAGAAGAGGAGGCTC

BACE1-F: GATCGCTAGCAGGAGTACAACTATGACAAGAGCATTGTG
BACE1-R: GATCTCTAGACGTGGAGGAGGCTGCCTTGATG

DNA oligonucleotides for RT-qPCR:
qEGFP-F: TCTGCACCACCGGCAAGCTG
qEGFP-R: TGCGCTCCTGGACGTAGCCT
qlaminC-F: AGATGATCCCTTGCTGACTTACCG
qlaminC-R: CCAGGTGTTCTGTGCCTTCCA
qP53-F: TAACAGTTCCTGCATGGGCGGC
qP53-R: AGGACAGGCACAAACACGCACC
qEGFR-F: GAAGAAGACATGGACGACG
qEGFR-R: TGGAATTGTTGCTGGTTG
qSCAP-F: ATTTGCTCACCGTGGAGATGTT
qSCAP-R: GAAGTCATCCAGGCCACTACTAATG
qFL-F: TCTAAGGAAGTCGGGGAAGC
qFL-R: CCCTCGGGTGTAATCAGAAT
qEXP5-F: GAACAGGAACTTCAGTTGGGAG
qEXP5-R: TGCGGCGGAGAGTATATAATGCA
qBeta-actin-F: CCCGCCGCCAGCTCACCAT
qBeta-actin-R: CATCGTCGCCCGCGAAGCC
qRPL7-F: GCTTCGATTAACATGCTGAGG
qRPL7-R: GCCATAACCACGCTTGTAGAT
qHNRNPD-F: GTGCTATTTAAAGAATCGGAGAGTG
qHNRNPD-R: GGCCCTTTTAGGATCAATCAC
qBEX2-F: ACCTCACGTCGAGAATCGGGA
qBEX2-R: CCTGGTTGACATTTTCCACGAT
qMSMO1-F: TTTGGGCATGGGTGACCATTC
qMSMO1-R: GGGATCAGATTTAAAGGGTTGAGA
qGAL-F: GCGCACAATCATTGAGTTTCTG
qGAL-R: AGGACCGCTCGATGTCTTCTGA
qKNSTRN-F: GGTTAAGACAGTGTATAGCCTGCAG
qKNSTRN-R: CATCGACTTCTTTGGACCTAACAGG
qMRPL35-F: CCACATCTGAGAGAAACCTGACATG
qMRPL35-R: CTGAAGTATGTTAGAGATCTGACTGGC

qDHTKD1-F: AGGCTGAATTTATTGACAGGCC
qDHTKD1-R: CAGGACGTCTCCAGTGGCTGAG
qMALSU1-F: CAACGAGGGACGCCCAGAAT
qMALSU1-R: CTTGCCTCAGAAGTGAAACCATC
qRAD1-F: TGGTATCAAAGTAACAGTGGAAAATGC
qRAD1-R: CGAAAAGTAACAGACTCTTCCTGAAC
qRPL31-F: CTTTGGTTACCTATGTACCTGTTACCAC
qRPL31-R: GATCTGACGATCAGCGATTAGTTC
qYWHAE-F: TCCTATTCGCTTAGGTCTTGCTCTC
qYWHAE-R: TATCCAGTTCTGCAATTGCATCAT
qACTR3-F: TCCAGAACAATCCTTGGAAACTG
qACTR3-R: CATTGATTCCAGTATACTGTTTAATCCA
qCCT4-F: CAGTCAGTGCTCTGACTCTTGCAAC
qCCT4-R: CCAGCCACAATACTGGTGATCATAA

DNA probe for Northern blotting with digoxigenin-labeled 3' end:
Anti-siGP: CGGCAAGCTGACCCTGAAGTT
Anti-siGPS: AACTTCAGGGTCAGCTTGCCG
Anti-sip53: TCCACTACAACTACATGTGTAT
Anti-siLC: GAACTGGACTTCCAGAAGAACT
Anti-U6 snRNA: ATTTGCGTGTCATCCTTGCGCAG

Synthetic RNA oligonucleotides:
shGP:
CGGCAAGCUGACCCUGAAGUUCUCGAGAACUUCAGGGUCAGCUUGCCGUU
saiGP:
AACUUCAGGGUCAGCUUGCCGUCAAGCUGACCCUGAAGUCAU

siGP-S: GCAAGCUGACCCUGAAGUUCA
siGP-AS: AACUUCAGGGUCAGCUUGCCG

shp53:
CCACUACAACUACAUGUGUAACUCGAGUUACACAUGUAGUUGUAGUGGUU
saip53:
UUACACAUGUAGUUGUAGUGGACUACAACUACAUGUGUAUAU

sip53-S: ACUACAACUACAUGUGUAACA
sip53-AS: UUACACAUGUAGUUGUAGUGG

siEXP5-1-S: CUCGAUUGGAGAAGGUGUATT
siEXP5-1-AS: UACACCUUCUCCAAUCGAGTT
siEXP5-2-S: UGCUCUGUCUCGAAUUGUATT
siEXP5-2-AS: UACAAUUCGAGACAGAGCATT

siEXP5-3-S: UGUGAGGAGGCAUGCUUGUTT

siEXP5-3-AS: ACAAGCAUGCCUCCUCACATT
